# Solvent and Copper Ion-Induced Synthesis of Pyridyl–Pyrazole-3-One Derivatives: Crystal Structure, Cytotoxicity

**DOI:** 10.3390/molecules22111813

**Published:** 2017-10-25

**Authors:** Qiu Ping Huang, Shao Nan Zhang, Shu Hua Zhang, Kai Wang, Yu Xiao

**Affiliations:** 1 Guangxi Key Laboratory of Electrochemical and Magnetochemical Functional Materials, Collaborative Innovation Center for Exploration of Hidden Nonferrous Metal Deposits and Development of New Materials in Guangxi (College of Chemistry and Bioengineering), Guilin University of Technology, Guilin 541004, China; huangqiupingclare@163.com (Q.P.H.); a591725185@163.com (S.N.Z.); kaiwang2011@yahoo.com (K.W.); 2 College of Chemistry and Engineering, Guangxi Normal University for Nationalities, Chongzuo 532200, China

**Keywords:** pyridyl–pyrazole-3-one derivatives, crystal structure, antitumor activity, in-situ reaction

## Abstract

Five novel compounds, methyl 5-(acetyloxy)-1-(6-bromo-2-pyridinyl)-1*H*-pyrazole-3-carboxylate (**1**), methyl 1-(6-bromo-2-pyridinyl)-5-hydroxy-1H-pyrazole-3-carboxylate (**2**), Trimethyl 1,1′,1′′-tris(6-bromo-2-pyridinyl)-5,5′′-dihydroxy-5′-oxo-1′,5′-dihydro-1H,1′′H-4,4′: 4′,4′′-terpyrazole-3,3′,3′′-tricarboxylate (H_2_L^1^, **3**), [Cu_2_(L^2^)_2_]·CH_3_OH (**4**), H_2_L^2A^·CH_3_CN (**5**) were synthesized. Compounds **1**–**5** characterized by elemental analysis, IR, and X-ray single-crystal diffraction. And **1**–**3** were also characterized by ^1^H NMR, ^13^C NMR and ESI-MS. The H_2_L^1^, H_2_L^2^ were formed by in-situ reaction. H_2_L^2^ and H_2_L^2A^ are mesomer compounds which have two chiral carbons. The antitumor activity of compounds **1**–**5** against BEL-7404, HepG2, NCI-H460, T-24, A549 tumor cell lines were screened by methylthiazolyl tetrozolium (MTT) assay. The compounds **1**, **2** showed weakly growth inhibition on the HepG2 cell lines. The HepG2 and A549 cell lines showed higher sensitivity to compound **4**, while the IC_50_ values are 10.66, 28.09 μM, respectively. It is worth noting that compounds **1**–**5** did not show cytotoxicity to human normal liver cell line HL-7702, suggesting its cytotoxic selectivity on these tumor cell lines.

## 1. Introduction

In the past two decades, metallodrugs have been widely applied in clinics to treat various diseases [1,2]. To date, the great successes achieved with platinum-based antitumour agents, such as cisplatin, carboplatin and oxaliplatin, have promoted the development of metal-based anticancer drugs [3,4,5,6,7]. However, all of the platinum-based drugs are associated with severe side effects and the evolution of drug resistance during therapy [8], which has further raised the researchers’ interests to synthesize and study non-platinum transition-metal complexes with satisfactory anticancer activities, less toxicities and specific antitumor mechanisms different to platinum-based anticancer drugs [9,10,11,12,13,14]. Copper, as an essential human element, has attracted many inorganic chemists to address copper(II) complexes with the aim for medical applications, due to their significant bioactivity and redox properties [15,16]. Copper can alter the metabolism of cancer cells and cause a differential response between normal and tumor cells. In recent years, copper coordination compounds have been considered good alternatives to platinum drugs as potential antitumour agents [17]. A number of synthetic copper(II) complexes have been reported as potential anticancer agents, active both in vitro and in vivo [18,19,20,21,22].

On the other hand, pyrazole derivatives are a subunit of many biologically active compounds. They have potential applications in medicinal chemistry as analgesics [23], therapeutic agents [24,25,26], antipyretics [27], antidepressants [28] and anti-inflammatories [29]. For example, 3-phenyl-imidazo[2,1-*b*]thiazol-6-one has recently exhibited increased potency towards the CNS SNB-75 and renal UO-31 cancer cell lines [30]. Moreover, the biological activities of pyrazole derivatives are influenced by electronegative radicals of substituted groups [31]. To further our search for new metal-based anticancer agents, we designed and synthesized four new pyrazole derivatives and the copper complex **4**. The in-vitro cytotoxicities of these compounds were evaluated by the MTT method.

## 2. Results and Discussion

### 2.1. Synthesis

**Scheme 1:**
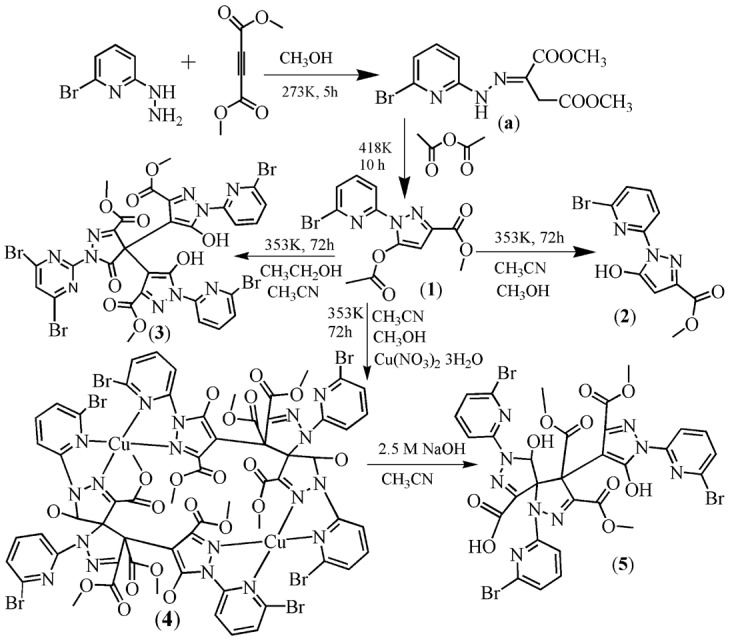
Synthetic routes for compounds **1**–**5**.

We aimed to synthesize novel pyridyl–pyrazole-3-one derivatives (Scheme 1). Compound **1** was prepared in a way similar to that in the literature, except that (6-chloro-pyridin-2-yl)-hydrazine was replaced by (6-bromo-pyridin-2-yl)-hydrazine [32]. Through a solvothermal method, compound **2** was synthesized, via one of the two aliphatic groups undergoing a hydrolysis reaction. However, when we carried out the reaction for **1** in mixed solvent (acetonitrile:ethanol, 8:7) under the solvothermal conditions, the trimeric complex **3** was obtained. Of course, the aliphatic groups also underwent a hydrolysis reaction. It is interesting to note that **3** is obviously different from **1** and **2**. On the basis of the synthetic conditions used to prepare **2** and **3**, it can be concluded that the solvent of the syntheses reactions is most likely responsible for the structure differences observed between **2** and **3**.

**Scheme 2:**
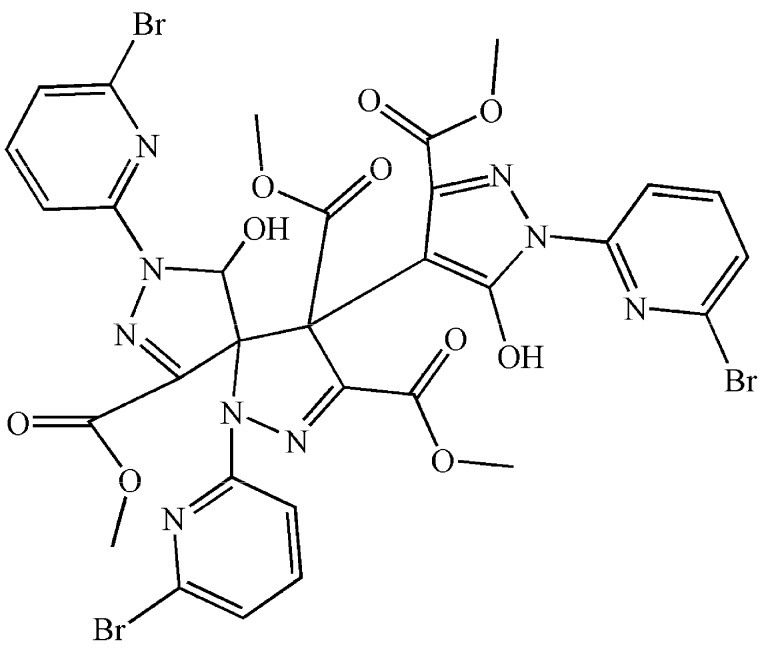
Molecular structure of H_2_L^2^.

In addition to the solvent effect, the metal ion also had an influence on the structure of the compounds. Having added the copper ion to the reaction system of **2**, a dinuclear copper complex [Cu_2_(L^2^)_2_]·(CH_3_OH) (**4**) was synthesized. H_2_L^2^, with two chiral centers, was synthesized by an in-situ reaction (Scheme 2). In order to understand the function of Cu(II) in the reaction, using Ni(II) or Co(II) ions to displace the Cu(II) ion meant that analogous complexes of **4** could not be obtained. The result indicates that the Cu(II) ion plays a key role in the formation of H_2_L^2^. In order to obtain H_2_L^2^, we slowly decreased the dosage of copper ion. At first, we still obtained **4**, but the yield was also decreased. When the molar ratio of Cu(NO_3_)_2_·3H_2_O:**1** decreased to 1:10, only compound **3** was obtained. Compound **5**, which is a derivative of HL^2^, can be obtained through a precipitation reaction (Equation (1)).

(1) $$[Cu{\left(L^{2}\right)}_{2}]\cdot CH_{3}OH+4H_{2}O\overset{NaOH}{\to }2H_{2}L^{2A}+CH_{3}OH+2Cu{\left(OH\right)}_{2}↓$$

### 2.2. Description of the Crystal Structures

#### 2.2.1. Crystal Structures of **1** and **2**

**Figure 1:**
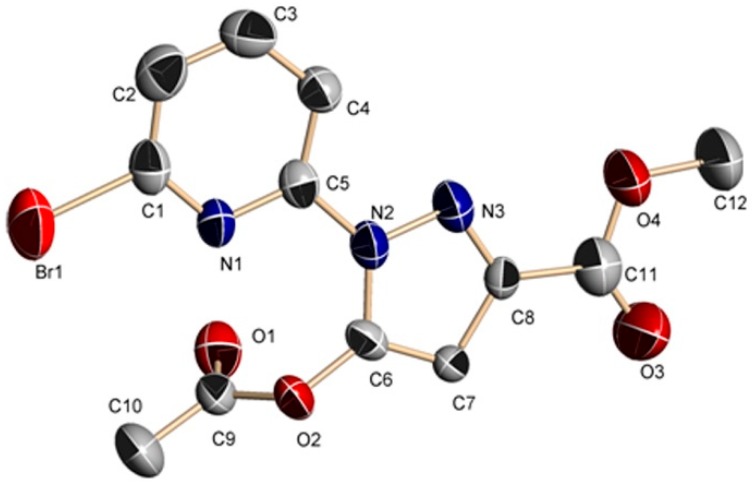
Molecular structure of **1**. Some hydrogen atoms were omitted for clarity.

**Figure 2:**
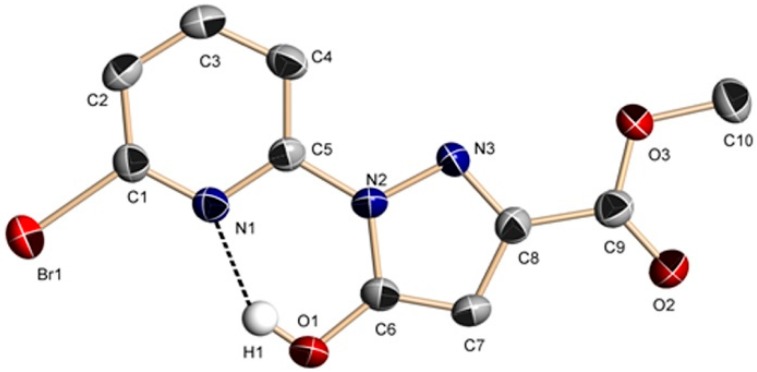
Molecular structure of **2**. Some hydrogen atoms were omitted for clarity.

The structures of complexes **1** and **2** are similar (Figure 1 and Figure 2, Tables S1 and S2). The substituent groups of the C6 atom are different. For **1** and **2**, the substituent groups of the C6 atom are acetate and hydroxyl groups, respectively. Therefore, only complex **1** is analyzed here.

Single-crystal X-ray diffraction analysis revealed that **1** belongs to the triclinic space group *P*ī with *a* = 8.230(1) Å, *b* = 8.282(1) Å, *c* = 10.214(1) Å, *α* = 75.47(1) Å, *β* = 85.27(1) Å, *γ* = 85.32(1) Å and *V* = 670.3(1) Å^3^. The molecular structure of **1** is shown in Figure 1. All bond distances in the pyrazole ring show partial double-bond character, which suggests a delocalized π-electronic system throughout the ring [33,34]. The bond angles and bond lengths (Table S1) in the pyrazole ring are within the normal ranges, close to the tabulated value [33]. The C6–C7 distance of 1.357 Å is consistent with a C=C double bond (average for C=C sp^2^–sp^2^ bond is 1.34 Å). The C7–C8 bond length of 1.408 Å is slightly shorter than the pure C–C sp^2^–sp^2^ single bond (average length for such a bond is 1.48 Å) [33]. The dihedral angle between the pyridine plane (–0.8294*x* + 0.4294*y* − 0.3574*z* = −1.7102) and the pyrazole plane (−0.7980*x* + 0.4102*y* − 0.4416*z* = −1.6966) is 5.271 degrees. The acetate carbonyl group substituent in position C6 is twisted from the plane of the pyrazole ring by –2.5 degrees (torsion angle C7–C8–C11–O3) and has cis orientation with respect to the C8–C7 bond. The compound **1** forms a 1D chain through intermolecular C–H···O hydrogen bonds (C2–H2···O1a, 3.400 Å, symmetry code: (a) *x*, −1 + *y*, *z,* Figure S1) which further constructs a 2D layer through C–H···O hydrogen bonds (C10–H10···O3b, 3.462 Å, symmetry code: (b) 1 − *x*, −1 − *y*, −*z*, Figure S2). The 2D layer forms a 3D network through intermolecular double Br···Br halogen bonds (Br1···Br1c, 3.856 Å, symmetry code: (c) 1 − *x*, −2 − *y*, −1 − *z*, Figure S3). The result of X-ray single-crystal analysis is consistent with that of NMR. It must be noted that the C6–O distances for **1** and **2** are 1.359 Å and 1.338 Å, respectively, which is close to the similar pyrazole–OH bond (1.329 Å) [34,35]. The result indicates that the bond C6–O is a C–O single bond. In addition, intramolecular O–H···N hydrogen bonds have been found in the crystal structure of **2**. The result of X-ray single crystal analysis is consistent with that of NMR.

#### 2.2.2. Crystal Structure of **3**

**Figure 3:**
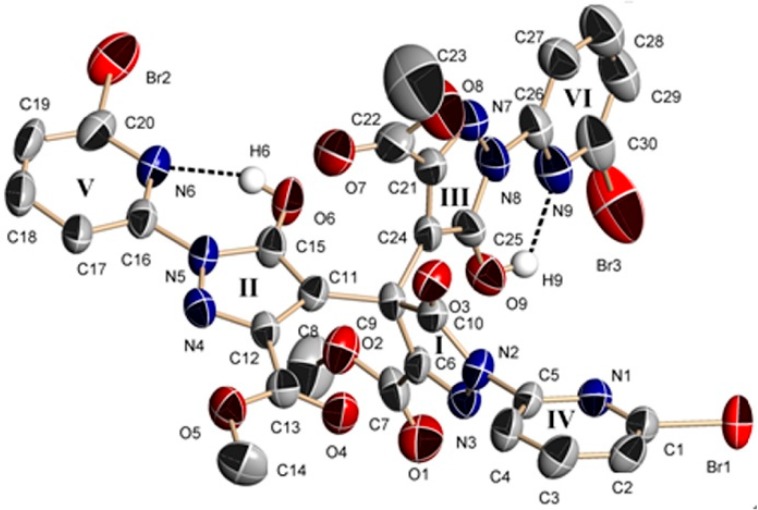
Molecular structure of **3**. Some hydrogen atoms were omitted for clarity.

Single-crystal X-ray diffraction analysis revealed that **3** belongs to the monoclinic space group *P*2_1_/*c* with *a* = 11.5817(12) Å, *b* = 12.3223(10) Å, *c* = 15.8773(11) Å, *α* = 83.661(6) Å, *β* = 71.060(8) Å, *γ* = 65.385(9) Å and *V* = 1947.7(3) Å^3^ (Figure 3). In **3**, all bond distances in the pyrazole rings (**II** and **III**) show partial double-bond character, which suggests a delocalized π-electronic system throughout the ring [34]. The bond angles and bond lengths (Table S3) in the pyrazole ring are within the normal ranges, close to the tabulated value [33]. The bond distances of C15–O6 and C25–O9 are 1.304 and 1.327 Å, respectively, which are close to the similar pyrazole–OH bond distance (1.329 Å) [32,35]. However, in the **I** ring, the C9 is sp^3^-hybridized. The bond distances of C9–C10, C9–C11, C9–C6 and C9–C24 are 1.541, 1.502, 1.519 and 1.549 Å, respectively, which are consistent with a C–C sp^3^–sp^2^ single bond (average length for C–C sp^3^–sp^2^ single bond is 1.51 Å) [36]. The bond distance of C10–O3 is 1.209 Å, which is obviously a carbonyl group (average length for C=O double bond is 1.20 Å) [36], while the C6–N3 distance is 1.248 Å, which is obviously a C=N double bond [33]. There are therefore no protons attached to N3 and O3. The rings of pyridyl–pyrazole (**II** and **V** rings) lie in the same plane, together with the substituents at **II** (O6 and ester carbonyl group) and at **V** (Br2), which is similar to the reported complex of 1-(6-chloro-pyridin-2-yl)-5-hydroxy-1*H*-pyrazole-3-carboxylic acid methyl ester [32]. The maximum deviation from the least-squares plane (–0.9382*x* + 0.2059*y* − 0.2780*z* = −13.6793) ranges from +0.1619 Å (C14) to −0.0914 Å (C18). However, the dihedral angle between the **III** and **VI** rings of pyridyl–pyrazole (least-squares plane equation: −0.1457*x* − 0.9464*y* − 0.1591*z* = −1.7409) and the plane of the ester carbonyl group (C22, C23, O7, O8, the least-squares plane equation: −0.1196*x* − 0.6125*y* − 0.7814*z* = −5.5691) is 45.2 degrees. It must be noted that the ring I is co-planar with the substituents at O3 and the methoxycarbonyl group (least-squares plane equation: 0.0826*x* + 0.3013*y* − 0.9500*z* = −2.7913). The dihedral angle between ring **I** with its substituents and the pyridine ring (ring **IV**) is 62.9 degrees. The compound **3** is constructed of a dimer through intermolecular double Br···Br halogen bonds (Br2···Br3a, 3.736Å, Br3···Br2a, 3.736Å, symmetry code: (a) 1 + *x*, *y*, *z*, Figure S4), and furthermore, forms a two-dimensional network through Br···O halogen bonds (Br1···O7b, 3.370Å, symmetry code: (b) *x*, *y* − 1, *z*; Figure S5). The result of X-ray single-crystal analysis is consistent with that of NMR.

#### 2.2.3. Crystal Structures of **4** and **5**

**Figure 4:**
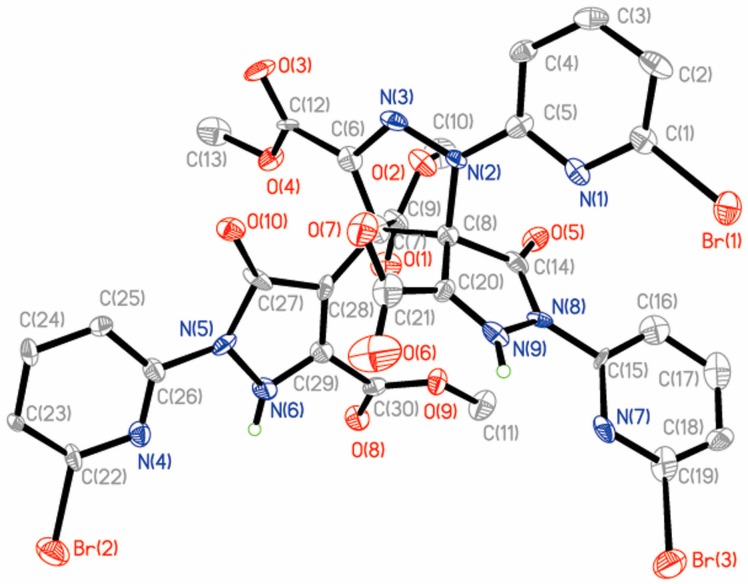
Molecular structure of **5**. Some hydrogen atoms were omitted for clarity.

Compound **5** (Figure 4) was obtained from **4**. The skeleton of H_2_L^2^^A^ is the same as H_2_L^2^ of **4** (Tables S4 and S5). **5** can be obtained through a precipitation reaction. Therefore, only complex **4** is analyzed here.

**Figure 5:**
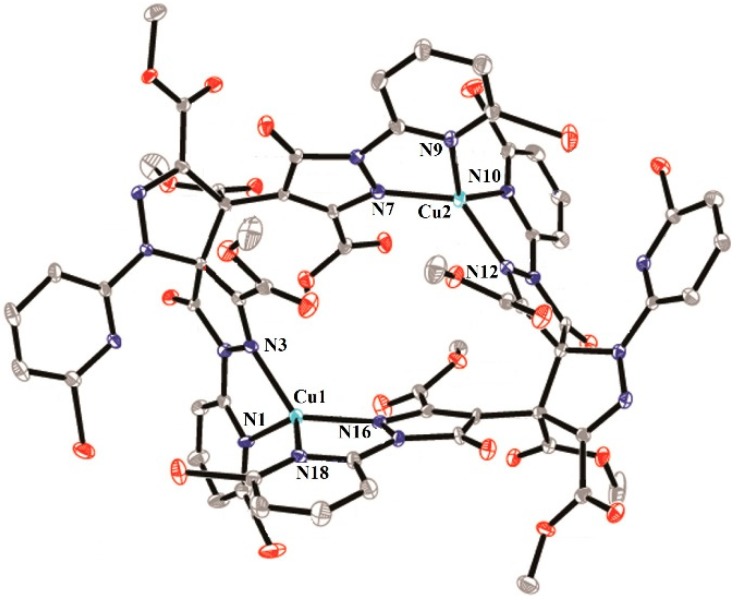
Molecular structure of **4**. Hydrogen atoms were omitted for clarity.

Single-crystal X-ray diffraction analysis reveals that **4** belongs to the monoclinic space group *P*2_1_/*c* with *a* = 14.3765(9) Å, *b* = 22.4070(13) Å, *c* = 23.662(2) Å, *β* = 96.982(7) Å and *V* = 7565.8(9) Å^3^. The dinuclear complex **4** is formed by two Cu(II) ions, two L^2^ ligands and one methanol solvent molecule (Figure 5). In **4**, the first Cu(II) ion (Cu1) is coordinated by four N atoms from two different L^2^ ligands, forming a distorted tetrahedron geometry. The bond distances of Cu1–N3, Cu1–N16, Cu1–N1 and Cu1–N18 are 2.026(8) Å, 1.979(8) Å, 2.105(8) Å and 2.078(9) Å, respectively (Table S4). Cu2 is also coordinated by four N atoms from two different L^2^ ligands, forming a distorted tetrahedron geometry. The bond distances of Cu2–N12, Cu1–N7, Cu1–N10 and Cu1–N9 are 2.004(8) Å, 1.976(9) Å, 2.073(9) Å and 2.064(9) Å, respectively. Two pyridyl–pyrazole rings of the L^2^ ligand bridge two Cu(II) ions to form a dinuclear compound. In the dimer, two Cu(II) ions and two L^2^ ligands form a pore, which shows approximate dimensions of 6.497 × 9.047 Å. Within this substructure, the Cu1···Cu2 distance is 6.497 Å and the C12···C43 distance is 9.047 Å. It is interesting to note that the four ester carbonyl groups may control the entry and exit of small molecules into the pore. It must be pointed out that the L^2^ ligand exists as a di-anion and displays a *μ*_2_:*η*^1^:*η*^1^:*η*^1^:*η*^1^ coordination mode. It is very interesting that the L^2^ ligand was synthesized by an in-situ reaction and formed two chiral centers (C11(*R*), C20(*R*) or C43(*R*), C52(*R*)). However, the complex **4** is not chiral, but is instead a meso molecule, possessing an axis of symmetry. The dinuclear complex **4** further forms a one-dimensional chain by intermolecular Br···Br (Br1···Br2a, 3.613 Å, symmetry code: (a) −*x*, −*y* + 2, −*z* + 1) and C–H···Br hydrogen bonds (Figure S6).

### 2.3. In-Vitro Antitumor-Activity Screening by MTT Assay

The cytotoxicities of compounds **1**–**5** against the human tumor cell lines BEL-7404, HepG2, NCI-H460, T-24 and A549, as well as human normal liver cell line HL-7702, were tested by the MTT assay. Cisplatin was also tested for comparison. As shown in Table S6, the inhibitory rates of **4** against BEL-7404, HePG2, T-24 and A549 are higher than that of **1**–**3** and **5**. **4** enhanced cytotoxicity and showed a synergetic effect after the ligand H_2_L^2^ coordinated with Cu(II) [37]. Moreover, **4** showed almost identical inhibitory rates against HepG2 to cisplatin.

The in-vitro cytotoxic activities of **1**–**5** and cisplatin were further investigated by determining the corresponding IC_50_ levels. As shown in Table 1, **4** exhibited lower IC_50_ values (10.66–42.89 μM) against BEL-7404, HepG2, A549 and T-24 than **1**–**3** and **5**. However, **3** exhibited a lower IC_50_ value (38.03 μM) against NCI-H460 than **4**. Notably, the IC_50_ value of **4** against HepG2 was 10.66 μM, which represented an approximately 16.44-fold increase compared to the free ligand **5** and a value equal to that of cisplatin (9.48 μM) [38]. The cytotoxicities of **1**–**4** toward the HepG2 tumor cells are enhanced by 2.98, 3.98, 2.76 and 9.02 times, respectively, compared with the normal liver cell HL-7702, indicating selective cytotoxicity of **1**–**4** to HepG2 cells. It is interesting to note that the cytotoxicities of **1**–**5** to normal liver cell HL-7702 are lower than that of cisplatin.

## 3. Experimental Section

### 3.1. Materials and Instrumentation

All chemicals were commercially available and used as received without further purification. The X-ray crystal structure was determined with an Agilent G8910A CCD (Palo Alto, CA, USA) diffractometer using the SHELXL crystallographic software (SHELXS-97, University of Gottingen, Göttingen, NI, Germany) for molecular structure. Elemental analyses (CHN) were performed using an Elemental Vario-EL CHN elemental analyzer (Elementar Analysensysteme, Hanau, MA, Germany). FT–IR spectra were recorded from KBr pellets in the range of 4000–400 cm^–1^ on a Bio-Rad FTS-7 spectrophotometer (Thermo Scientific, Waltham, MA, USA). The ^1^H- and ^13^C-NMR spectra were recorded on a Bruker AV500 spectrometer (Varian, Palo Alto, CA, USA), using tetramethylsilane (TMS) as internal standard and CDCl_3_ as solvent. ESI–MS spectra were recorded on a Bruker HTC Electrospray Ionization Mass Spectrometer. MCO96 (Sanyo electric co., LTD, Osaka, Japan); HB-402V (Hanbeak corporation, Seoul, Korea); PM-10AK (Olympus corporation, Tokyo, Japan); ELX 800 (Bio Tek instruments, Winooski, VT, USA). Five human tumor cell lines, BEL-7404, HepG2, NCI-H460, T-24 and A549, and the normal liver cell line HL-7702, were all obtained from the Institute of Biochemistry and Cell Biology, Chinese Academy of Sciences (The cell bank, Chinese academy of sciences, Shanghai, China).

### 3.2. Synthesis

*Methyl 5-(acetyloxy)-1-(6-bromo-2-pyridinyl)-1H-pyrazole-3-carboxylate* (**1**). Dimethyl acetylenedicarboxylate (DMADC) (6.00 mL, 48.9 mmol) in methanol (60 mL) was added dropwise to a solution of (6-bromo-pyridin-2-yl)-hydrazine (6.50 g, 34.8 mmol) in methanol (50 mL) and the mixture was stirred vigorously for 5 h at 0 °C. The resulting suspension was filtered and the filter cake was washed thoroughly with cold methanol. The filter cake was recrystallized from acetone to obtain the intermediate product 2-[(6-Bromo-pyridin-2-yl)-hydrazono]-succinic acid dimethyl ester (**a**, 7.63 g, 72%) as a yellow solid.

A solution of **a** (5.78 g, 17.5 mmol) in acetic anhydride (60 mL) was heated under reflux for 10 h. The resulting solution was evaporated to dryness, methanol (12 mL) was added, and the suspension was cooled to 10 °C and filtered. Using methanol as solvent, **1** was obtained through recrystallization. Yield: 3.54 g (68% based on (6-bromo-pyridin-2-yl)-hydrazine). *Anal. Calc*. for **1**: C_12_H_10_N_3_O_4_Br (*M*_r_ = 340.14), calc.: C, 42.37%; H, 2.96%; N, 12.35%; *found*: C, 47.31%; H, 2.99%; N, 12.38%. ^1^H-NMR (CDCl_3_) δ 2.48 (s, 3H), 3.97 (s, 3H), 6.66 (s, 1H), 7.45 (d, *J* = 8.0 Hz, 1H), 7.69–7.72 (m, 1H), 7.99 (d, *J* = 8.0 Hz, 1H) ppm. ^13^C-NMR (CDCl_3_) δ 20.7, 52.4, 101.3, 114.1, 126.7, 139.3, 140.7, 143.7, 146.0, 151.0, 161.9, 167.7 ppm. ESI–MS (MS^–^): 338. IR data for **1** (KBr, cm^–1^: 3415, 1789, 1718, 1576, 1427, 1378, 1250, 1179, 1100, 1058, 1015, 937, 874, 795, 746, 661, 596. IR, ^1^H-NMR, ^13^C-NMR and ESI–MS spectra of compound **1** are shown in Figures S7–S10, respectively.)

*Methyl 1-(6-bromo-2-pyridinyl)-5-hydroxy-1H-pyrazole-3-carboxylate* (**2**). A mixture of **1** (0.160 g, 0.5 mmol), acetonitrile (5 mL) and methanol (5 mL) was put into a Teflon-lined autoclave (15 mL) and then heated at 80 °C for 3 days. White crystals of **2** were collected by filtration, washed with methanol and dried in air. Pure crystals of **2** were obtained by manual separation. Yield: 0.108 g (86% based on **1**). Anal. Calc. for **2**: C_10_H_8_N_3_O_3_Br (*M*_r_ = 297.12), calc.: C, 40.42%; H, 2.71%; N, 14.14%; found: C, 40.38%; H, 2.75%; N, 14.16%. ^1^H-NMR (CDCl_3_) δ 3.94 (s, 3H), 6.11 (s, 1H), 7.43 (d, *J* = 7.5 Hz, 1H), 7.76–7.79 (m, 1H), 8.03 (d, *J* = 8.0 Hz, 1H), 11.36 (s, 1H) ppm. ^13^C-NMR (CDCl_3_) δ 52.3, 90.6, 111.7, 125.4, 137.5, 141.9, 144.9, 153.7, 156.2, 162.4 ppm. ESI–MS (MS^–^): 296. IR data for **2** (KBr, cm^–1^): 3437, 3146, 2358, 1718, 1590, 1442, 1257, 1108, 1001, 937, 803, 710, 441. IR, ^1^H-NMR, ^13^C-NMR and ESI–MS spectra of compound **2** are shown in Figures S11–S14, respectively.)

*Trimethyl 1,1′,1′′-tris(6-bromo-2-pyridinyl)-5,5′′-dihydroxy-5′-oxo-1′,5′-dihydro-1H,1′′H-4,4′: 4′,4′′-terpyrazole-3,3′,3′′-tricarboxylate (H_2_L^1^)* (**3**). A mixture of **1** (0.160 g, 0.5 mmol), acetonitrile (8 mL) and ethanol (7 mL) was put into a Teflon-lined autoclave (25 mL) and then heated at 80 °C for 3 days. White crystals of **3** were collected by filtration, washed with methanol and dried in air. Pure crystals of **3** were obtained by manual separation. Yield: 0.058 g (51**%** based on **1**). Anal. Calc. for **3**: C_30_H_20_Br_3_N_9_O_9_ (*M*_r_ = 890.25), calc: C, 40.47%; H, 2.26%; N, 14.15%; found: C, 40.36%; H, 2.40%; N, 14.11%. ^1^H-NMR (CDCl_3_) δ 3.73 (s, 6H), 3.94 (s, 3H), 7.28 (s, 1H), 7.38 (d, *J* = 7.5 Hz, 1H), 7.44 (d, *J* = 7.5 Hz, 2H), 7.60–7.63 (m, 1H), 7.78–7.81 (m, 2H), 7.92 (d, *J* = 8 Hz, 1H), 7.80 (d, *J* = 8.5 Hz, 1H) , 8.05 (d, *J* = 8.0 Hz, 1H), 11.77 (s, 1H) ppm. ^13^C-NMR (CDCl_3_) δ 29.7, 52.3, 52.6, 52.6, 58.5, 95.0, 97.6, 111.5, 111.9, 113.7, 125.6, 125.8, 125.8, 137.3, 137.4, 139.9, 140.5, 141.8, 142.0, 145.0, 149.2, 149.5, 152.7, 153.3, 153.6, 156.1, 160.5, 162.3, 162.5, 172.2 ppm. ESI–MS (MS^–^): 889. IR data for **3** (KBr, cm^–1^): 3415, 2371, 1738, 1619, 1442, 1314, 1243, 1122, 909, 795, 625, 476. IR, ^1^H-NMR, ^13^C-NMR and ESI–MS spectra of compound **3** are shown in Figures S15–S18, respectively.

*Trimethyl-1,8-bis(6-bromopyridin-2-yl)-4-(1-(6-bromopyridin-2-yl)-5-hydroxy-3-(methoxycarbonyl)-1H-pyrazol-4-yl)-9-hydroxy-1,2,7,8-tetraazaspiro[4.4]nona-2,6-diene-3,4,6-tricarboxylate ([Cu_2_(L^2^)_2_]·CH_3_OH)* (**4**). A mixture of Cu(NO_3_)_2_·3H_2_O (0.120 g, 0.5 mmol), **1** (0.148 g, 0.5 mmol), acetonitrile (8 mL) and methanol (7 mL) was poured into a Teflon-lined autoclave (25 mL) and then heated at 80 °C for 3 days. Red crystals of **4** were collected by filtration, washed with methanol and dried. Pure crystals of **4** were obtained by manual separation. Yield: 0.074 g (45% based on **1**). Anal. Calc. for **4**: C_6__3_H_4__6_Cu_2_N_18_O_2__1_Br_6_ (*M*_r_ = 1997.72), calc: C, 37.87%; H, 2.32%; N, 12.61%; found: C, 37.81%; H, 2.39%; N, 12.69%. IR data for **4** (KBr, cm^–1^, Figure S19): 3451, 1732, 1633, 1406, 1307, 1115.

*1,8-Bis(6-bromopyridin-2-yl)-4-(1-(6-bromo pyridin-2-yl)-5-hydroxy-3-(methoxycarbonyl)-1H-pyrazol-4-yl)-9-hydroxy-3,4-bis(methoxycarbonyl)-1,2,7,8-tetraazaspiro[4.4]nona-2,6-diene-6-carboxylic acid (H_2_L^2^^A^·CH_3_CN)* (**5**). A solution of **4** (0.1 mmol, 0.1995 g) in acetonitrile (15 mL) was treated with NaOH solution (5 mL, 2.5 mol/L). The mixture was stirred for 3 h and filtered. The filtrate was left for 15 d. White and block single crystals of **5** were collected by filtration, washed with acetonitrile and dried in air. Yield: 0.058 g (80% based on **4**). Anal. Calc. for **5**: C_3__3_H_2__6_Br_3_N_10_O_10_ (*M*_r_ = 962.37), calc: C, 41.18%; H, 2.72%; N, 14.55%; found: C, 41.11%; H, 2.79%; N, 14.48%. IR data for **5** (KBr, cm^–1^, Figure S20): 3433, 1734, 1587, 1441, 1294, 1116, 1021, 779, 715.

### 3.3. X-ray Crystallography

Five diffraction datasets were collected on an Agilent G8910A CCD diffractometer with graphite monochromated Mo-K*α* radiation (*λ* = 0.71073 Å) using the *ω*-*θ* scan mode in the ranges 3.13° ≤ *θ* ≤ 26.37° (**1**), 2.91° ≤ *θ* ≤ 26.37° (**2**), 2.95° ≤ *θ* ≤ 25.01° (**3**), 2.85° ≤ *θ* ≤ 26.37° (**4**) and 3.27° ≤ *θ* ≤ 25.00° (**5**). Raw frame data were integrated with the SAINT program. The structures were solved by direct methods using SHELXS-97 and refined by full-matrix least-squares on *F*^2^ using SHELXS-97 [39]. An empirical absorption correction was applied with the program CrysAlis RED (Agilent, 2012). All non-hydrogen atoms were refined anisotropically. All hydrogen atoms were positioned geometrically and refined as riding. Calculations and graphics were performed with SHELXTL [39]. The highest peak and deepest hole of **3** in the residual electron density are located 0.81 Å from atom O10 and 1.45 Å from atom O10, respectively. The highest peak and deepest hole of **4** in the residual electron density are located 3.05 Å from atom H61 and 0.95 Å from atom Br3, respectively. Selected bond distances and angles of the compounds **1**–**5** are listed in Tables S1–S5. The crystallographic details are provided in Table S7. Crystallographic data for the structural analysis have been deposited with the Cambridge Crystallographic Data Center (CCDC reference numbers: 1532795–1532799).

### 3.4 Cell Culture and Treatment

All the cells were maintained in DMEM (Dulbecco’s Modified Eagle Medium) supplemented with 10% fetal calf serum, 100 units/mL ampicillin and 100 mg/mL streptomycin sulfate at 37 °C in a humidified atmosphere under 5% CO_2_. Compounds **1**–**5** were dissolved in DMSO at a concentration of 2.0 mM stock solution. The stock was diluted by PBS to the required concentration immediately before use.

### 3.5. In-Vitro Cytotoxicity MTT Assay

Cisplatin was prepared according to a literature procedure [38]. The cells were all grown in 96-well, flat-bottomed microtiter plates. Compounds **1**–**5** and cisplatin were dissolved in the culture medium (containing 1% DMSO) to a concentration of 100 μg/mL. The resultant solutions were subsequently added to a set of wells. The wells for control contained the supplemented medium with 1% DMSO. The microtiter plates were incubated at 37 °C in a humidified atmosphere of 5% CO_2_/95% air for a further 48 h. At the end of each incubation period, the 3-(4,5-dimethylthiazol-2-yl)-2,5-diphenyltetrazolium bromide (MTT) solution (10 μL, 5 mg/mL) was added into each well and the cultures were further incubated for 6 h under the same conditions. After the removal of the supernatant, DMSO (100 μL) was added to dissolve the formazan crystals. The absorbance of the solution was read by an enzyme-labeled microplate reader with 490/630 nm double wavelength measurement. The cytotoxicity was evaluated based on the percentage of cell survival in a dose-dependent manner relative to the negative control. The final IC_50_ values were calculated by the Bliss method (*n* = 5). All tests were repeated in at least three independent trials.

## 4. Conclusions

Five novel pyridyl–pyrazole-3-one derivatives have been synthesized. H_2_L^1^ and H_2_L^2^ were formed by an in-situ reaction. Compound **5** was obtained from **4**. H_2_L^2^ and H_2_L^2A^ are meso compounds that have two chiral carbons. The result indicates that the Cu(II) ion and the solvent may play key roles in the formation of **2**–**4**. The in-vitro cytotoxicities of **1**–**5** against five selected human tumor cell lines and a normal cell line are different. **4** exhibited lower IC_50_ values against BEL-7404, HepG2, A549 and T-24 than did **1**–**3** and **5**. However, **2** and **3** exhibited lower IC_50_ value against NCI-H460 than did **4**. It is interesting to note that **1**–**4** have selective cytotoxicity to HepG2 cells.

## Figures and Tables

**Table 1:** IC_50_^a^ (μM) values of **1**–**5** and cisplatin on the selected cells for 48 h.

Compounds	BEL-7404	HepG2	NCI-H460	T-24	A549	HL-7702
**1**	66.51 ± 1.13	37.15 ± 0.54	108.97 ± 1.65	70.51 ± 1.33	37.56 ± 1.03	110.65 ± 2.84
**2**	125.20 ± 1.87	29.75 ± 0.91	40.67 ± 0.54	46.21 ± 0.87	44.77 ± 1.12	118.36 ± 3.34
**3**	112.28 ± 2.78	54.74 ± 0.81	38.03 ± 0.89	85.38 ± 0.67	33.56 ± 1.07	150.83 ± 3.06
**4**	41.81 ± 0.37	10.66 ± 0.38	66.48 ± 0.57	42.89 ± 1.41	28.09 ± 1.01	96.14 ± 0.49
**5**	84.26 ± 1.01	175.23 ± 1.10	94.56 ± 0.41	76.03 ± 1.16	77.56 ± 0.72	102.26 ± 0.85
Cisplatin ^b^	12.41 ± 0.38	9.48 ± 0.35	18.89 ± 1.02	28.07 ± 1.88	9.48 ± 0.35	5.63 ± 0.32

**^a^** IC_50_ values are presented as the mean ± SD (standard error of the mean) from five independent experiments. **^b^** Cisplatin was dissolved to a concentration of 1 mM in 0.154 M NaCl [38].

## Supplementary Materials

Supplementary materials are available online. CCDC 1532795-1532798 and 1541597 contain the supplementary crystallographic data for compounds **1**–**5**. These data can be obtained free of charge from the Cambridge Crystallographic Data Centre via http://www.ccdc.cam.ac.uk/data_request/cif. Electronic Supplementary Information (ESI) available: 1D chain of **1**. 2D layer of **1**. The IR spectra of **1**–**5**. The dimer structure of **3**. The packing drawings of **1**–**4**. Bond lengths and bond angles of **1**–**5**. The inhibition rates of **1**–**5** towards six selected cell lines. The ^1^H-NMR, ^13^C-NMR and ESI–MS spectra of **1**–**3**.

## References

[1] Milacic V., Chen D., Ronconi L., Landis-Piwowar K.R., Fregona D., Dou Q.P. (2006). A Novel Anticancer Gold(III) Dithiocarbamate Compound Inhibits the Activity of a Purified 20S Proteasome and 26S Proteasome in Human Breast Cancer Cell Cultures and Xenografts. Cancer Res..

[2] Kostova I. (2006). Gold coordination complexes as anticancer agents. Anticancer Agents Med. Chem..

[3] Jung Y., Lippard S.J. (2007). Direct cellular responses to platinum-induced DNA damage. Chem. Rev..

[4] Bruijnincx P.C.A., Sadler P.J. (2008). New trends for metal complexes with anticancer activity. Chem. Biol..

[5] Hartinger C.G., Nazarov A.A., Ashraf S.M., Dyson P.J., Keppler B.K. (2008). Carbohydrate-metal complexes and their potential as anticancer agents. Curr. Med. Chem..

[6] Richardson D.R., Kalinowski D.S., Richardson V., Philip C.S., David B.L., Mohammad I., Bernhardt P.V. (2009). 2-Acetylpyridine thiosemicarbazones are potent iron chelators and antiproliferative agents, redox activity, iron complexation and characterization of their antitumor activity. J. Med. Chem..

[7] Hemmert C., Fabié A., Fabre A., Françoise B., Heinz G. (2013). Synthesis, structures, and antimalarial activities of some silver(I), gold(I) and gold(III) complexes involving N-heterocyclic carbene ligands. Eur. J. Med. Chem..

[8] Bielefeld E.C., Tanaka C., Coling D., Li M., Henderson D., Fetoni A.R. (2013). An Src-protein tyrosine kinase inhibitor to reduce cisplatin ototoxicity while preserving its antitumor effect. Anti-Cancer Drugs.

[9] Rosenberg B., Vancamp L., Trosko J.E., Mansour V.H. (1969). Platinum compounds, a new class of potent antitumour agent. Nature.

[10] Barry N.P.E., Sadler P.J. (2013). Exploration of the medical periodic table, towards new targets. Chem. Commun..

[11] Yadav A., Janaratne T., Krishnan A., Singhal S.S., Yadav S., Dayoub A.S., Hawkins D.L., Awasthi S., MacDonnell F.M. (2013). Regression of lung cancer by hypoxia-sensitizing ruthenium polypyridyl complexes. Mol. Cancer Ther..

[12] Zhang H.Y., Wang W., Chen H., Zhang S.H., Li Y. (2016). Five novel dinuclear copper(II) complexes, Crystal structures, properties, Hirshfeld surface analysis and vitro antitumor activity study. Inorg. Chim. Acta.

[13] Christian G.H., Andrew D.P., Alexey A.N. (2011). Polynuclear ruthenium, osmium and gold complexes. The quest for innovative anticancer chemotherapeutics. Curr. Top. Med. Chem..

[14] Chen Z.F., Gu Y.Q., Song X.Y., Peng Y., Liang H. (2013). Synthesis, crystal structure, cytotoxicity and DNA interaction of 5,7-dichloro-8-quinolinolato-lanthanides. Eur. J. Med. Chem..

[15] Green D.R., Reed J.C. (1998). Mitochondria and apoptosis. Science.

[16] Easmon J., Pürstinger G., Heinisch G., Roth T., Fiebig H.H., Holzer W., Jager W., Jenny M., Hofmann J. (2001). Synthesis, cytotoxicity, and antitumor activity of copper(II) and iron(II) complexes of 4 N-azabicyclo [3.2.2] nonane thiosemicarbazones derived from acyl diazines. J. Med. Chem..

[17] Griffith D., James P.P., Celine J.M. (2010). Enzyme inhibition as a key target for the development of novel metal-based anti-cancer therapeutics. Anti-Cancer Agents Chem..

[18] O’Connor M., Kellett A., McCann M., Rosair G., McNamara M., Howe O., Creaven B.S., McClean S., Kia A.F., O’Shea D. (2012). Copper(II) complexes of salicylic acid combining superoxide dismutase mimetic properties with DNA binding and cleaving capabilities display promising chemotherapeutic potential with fast acting in vitro cytotoxicity against cisplatin sensitive and resistant cancer cell lines. J. Med. Chem..

[19] Tardito S., Barilli A., Bassanetti I., Tegoni M., Bussolati O., Franchi-Gazzola R., Mucchino C., Marchio L. (2012). Copper-dependent cytotoxicity of 8-hydroxyquinoline derivatives correlates with their hydrophobicity and does not require caspase activation. J. Med. Chem..

[20] Palanimuthu D., Shinde S.V., Somasundaram K., Samuelson A.G. (2013). In vitro and in vivo anticancer activity of copper bis (thiosemicarbazone) complexes. J. Med. Chem..

[21] Raja D.S., Bhuvanesh N.S.P., Natarajan K. (2011). Biological evaluation of a novel water soluble sulphur bridged binuclear copper (II) thiosemicarbazone complex. Eur. J. Med. Chem..

[22] Manikandamathavan V.M., Parameswari R.P., Weyhermüller T., Vasanthi H.R., Nair B.U. (2011). Cytotoxic copper(II) mixed ligand complexes, Crystal structure and DNA cleavage activity. Eur. J. Med. Chem..

[23] Gürsoy A., Demirayak Ş., Çapan G., Erol K., Vural K. (2000). Synthesis and preliminary evaluation of new 5-pyrazolinone derivatives as analgesic agents. Eur. J. Med. Chem..

[24] Jiang J.B., Hesson D.P., Dusak B.A., Dexter G.J., Hamel E. (1990). Synthesis and biological evaluation of 2-styrylquinazolin-4 (3*H*)-ones, a new class of antimitotic anticancer agents which inhibit tubulin polymerization. J. Med. Chem..

[25] Mohareb R.M., El-Sayed N.N.E., Abdelaziz M.A. (2012). Uses of cyanoacetylhydrazine in heterocyclic synthesis, novel synthesis of pyrazole derivatives with anti-tumor activities. Molecules.

[26] Anzai K., Furuse M., Yoshida A., Matsuyama A., Moritake T., Tsuboi K., Ikota N. (2004). In vivo radioprotection of mice by 3-methyl-1-phenyl-2-pyrazolin-5-one (Edaravone; Radicut^®^), a clinical drug. J. Radiat. Res..

[27] Soad A.M., Hawash E.L., Sayed E.L., Badawey A.M., Ibrahim M., Ashmawey E.I. (2006). El–Ashmawey Nonsteroidal antiinflammatory agents-part 2 antiinflammatory, analgesic and antipyretic activity of some substituted 3-pyrazolin-5-ones and 1,2,4,5,6,7-3*H*-hexahydroindazol-3-ones. Eur. J. Med. Chem..

[28] Bailey D.M., Hansen P.E., Hlavac A.G., Baizman E.R., Pearl J., DeFelice A.F., Feigenson M.E. (1985). 3,4-Diphenyl-1*H*-pyrazole-1-propanamine antidepressants. Eur. J. Med. Chem..

[29] Badawey E.S.A.M., El-Ashmawey I.M. (1998). Nonsteroidal antiinflammatory agents-Part 1, Antiinflammatory, analgesic and antipyretic activity of some new 1-(pyrimidin-2-yl)-3-pyrazolin-5-ones and 2-(pyrimidin-2-yl)-1,2,4,5,6,7-hexahydro-3*H*-indazol-3-ones. Eur. J. Med. Chem..

[30] Ali A.R., El-Bendary E.R., Ghaly M.A., Shehata I.A. (2014). Synthesis, in vitro anticancer evaluation and in silico studies of novel imidazo[2,1-*b*]thiazole derivatives bearing pyrazole moieties. Eur. J. Med. Chem..

[31] Das N., Verma A., Shrivastava P.K., Shravastava S.K. (2008). Synthesis and biological evaluation of some new aryl pyrazol-3-one derivatives as potential hypoglycemic agents. Indian J. Chem. Technol..

[32] Shen L.Q., Huang S.Y., Diao K.S., Lei F.H. (2012). Synthesis, crystal structure, computational study of 1-(6-chloro-pyridin-2-yl)-5-hydroxy-1*H*-pyrazole-3-carboxylic acid methyl ester and its 5-acetoxy analogs. J. Mol. Struct..

[33] Allen F.H., Davies J.E., Galloy J.J., Johnson O., Kennard O., Macrae C.F., Mitchelle M., Mitchell G.F., Smith J.M., Watson D.G. (1991). The development of versions 3 and 4 of the Cambridge Structural Database System. J. Chem. Inf. Comp. Sci..

[34] Dimova V., Jordanov I., Dimitrov L. (2016). Uv Spectrophotometric Determination OF pK′s OF 1,2,4-triazoline-3-thiones IN sodium hydroxide solutions. J. Chil. Chem. Soc..

[35] Ciolkowski M., Paneth P., Lorenz I.P., Rozalski M., Krajewska U., Budzisz E. (2009). Tautomeric forms study of 1*H*-(2′-pyridyl)-3-methyl-5-hydroxypyrazole and 1*H*-(2′-pyridyl)-3-phenyl-5-hydroxypyrazole. Synthesis, structure, and cytotoxic activity of their complexes with palladium (II) ions. J. Enzym. Inhib. Med. Chem..

[36] Carey F.A., Sundberg R.J. (2007). Advanced Organic Chemistry, Part A, Structure and Mechanisms.

[37] Zou H.H., Wang L., Long Z.X., Qin Q.P., Song Z.K., Xie T., Zhang S.H., Liu Y.C., Lin B., Chen Z.F. (2016). Preparation of 4-([2,2′,6′,2″-terpyridin]-4′-yl)-*N*,*N*-diethylaniline Ni II and Pt II complexes and exploration of their in vitro cytotoxic activities. Eur. J. Med. Chem..

[38] Cao R., Jia J., Ma X., Zhou M., Fei H. (2013). Membrane localized iridium(III) complex induces endoplasmic reticulum stress and mitochondria-mediated apoptosis in human cancer cells. J. Med. Chem..

[39] Sheldrick G.M. (2015). Crystal structure refinement with SHELXL. Acta Cryst..

