# Supplementary material for: Solvent and Copper Ion-Induced Synthesis of Pyridyl–Pyrazole-3-One Derivatives: Crystal Structure, Cytotoxicity
Source: Molecules. 2017 Oct 25;22(11):1813. doi: 10.3390/molecules22111813 (PMC6150270; doi:10.3390/molecules22111813)
Supplement: Supplementary file 1 [file molecules-22-01813-s001.pdf]

Solvent and Copper Ion Induced SYNTHESIS

Pyridyl-Prazol-3-one Derivatives: Crystal Structure, Cytotoxicity

Qiu Ping Huang [1,2,†], Shao Nan Zhang [1,†], Shu Hua Zhang [1,*], Kai Wang [1] and Yu Xiao [1,*]

1 Guangxi Key Laboratory of Electrochemical and Magnetochemical Functional Materials, Collaborative Innovation Center for Exploration of Hidden Nonferrous Metal Deposits and Development of New Materials in Guangxi (College of Chemistry and Bioengineering), Guilin University of Technology, Guilin 541004, China; huangqiupingclare@163.com (Q.P.H.); a591725185@163.com (S.N.Z.); kaiwang2011@yahoo.com (K.W.)

2 College of Chemistry and Engineering, Guangxi Normal University for Nationalities, Chongzuo 532200, China

* Correspondence: zsh720108@163.com (S.H.Z.); xiaoyu0926@sohu.com (Y.X.); Tel./Fax: +86-773-253-8340 (S.H.Z. & Y.X.);

† Contributed equally to this work

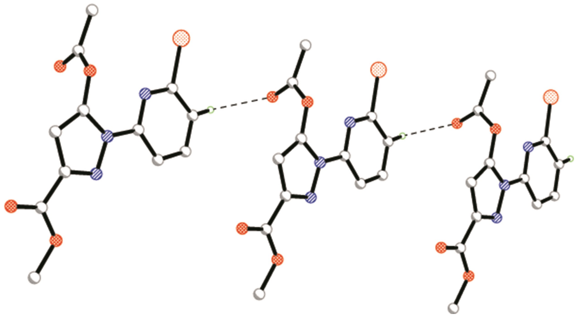

Figure S1. 1D chain of **1**.

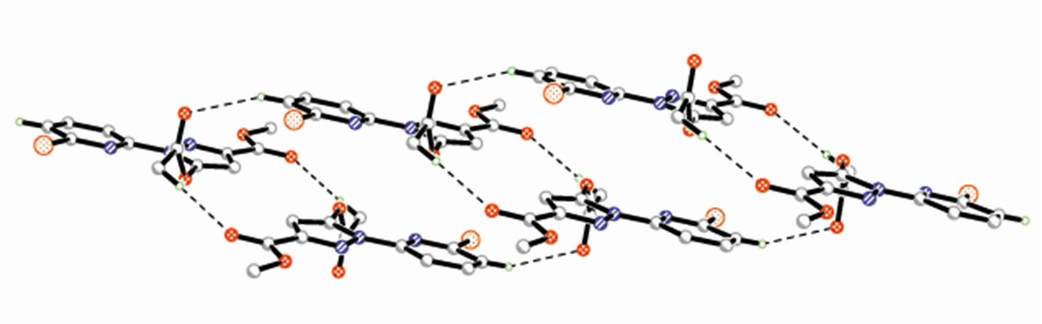

Figure S2. 2D layer of **1**.

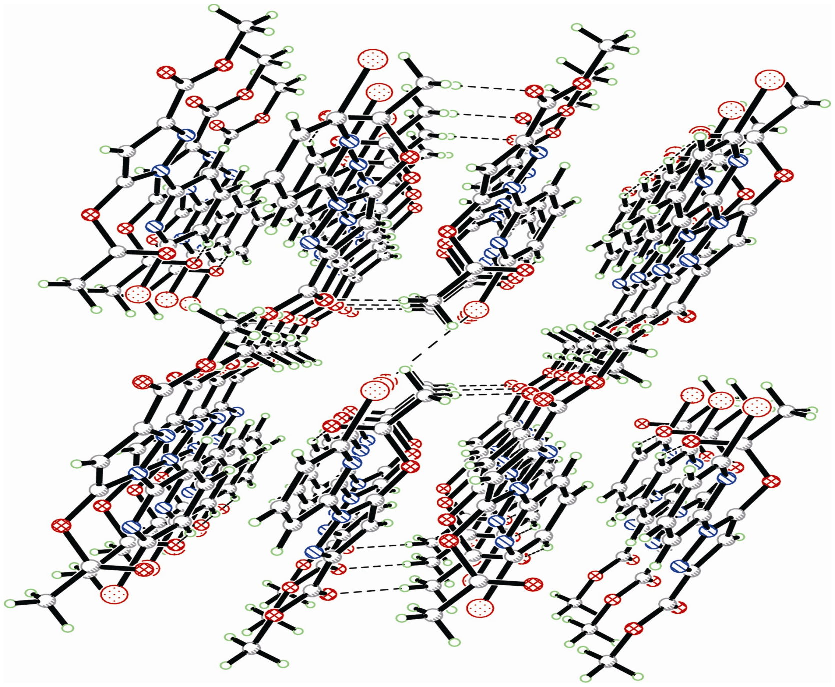

Figure S3. Packing drawing of **1**.

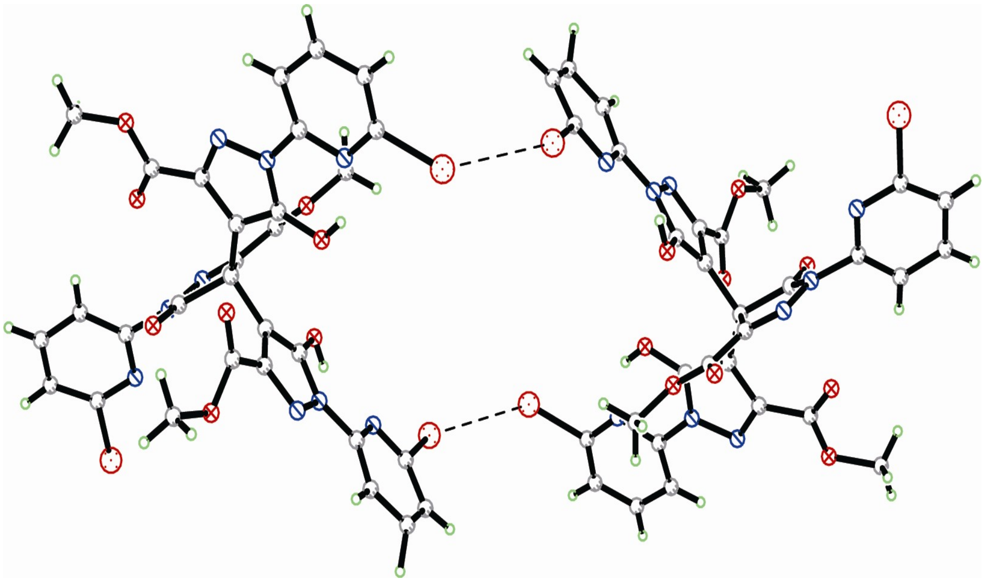

Figure S4.The dimer structure of **3**.

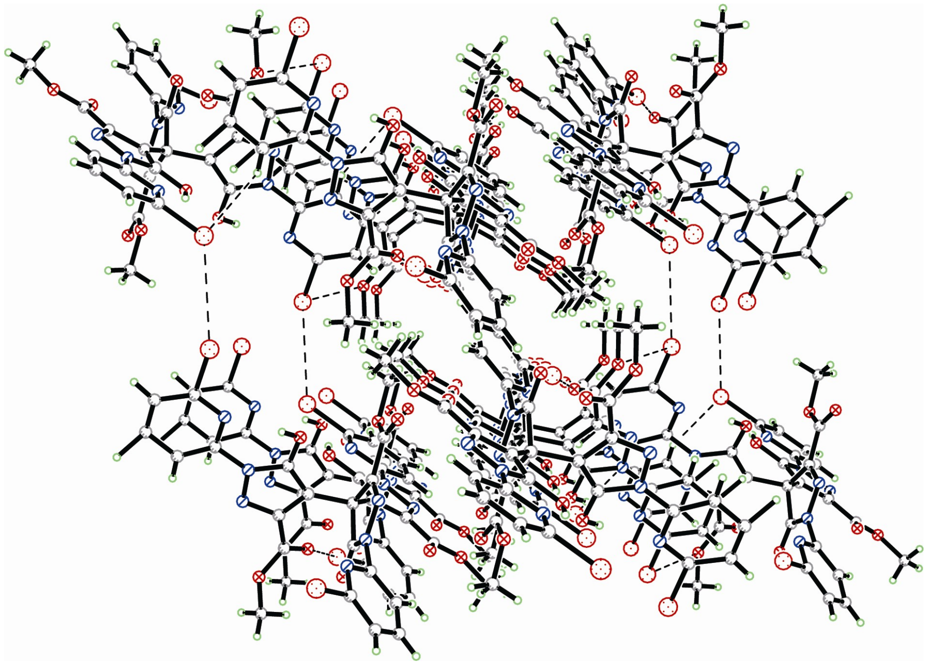

Figure S5. Packing drawing of **3**.

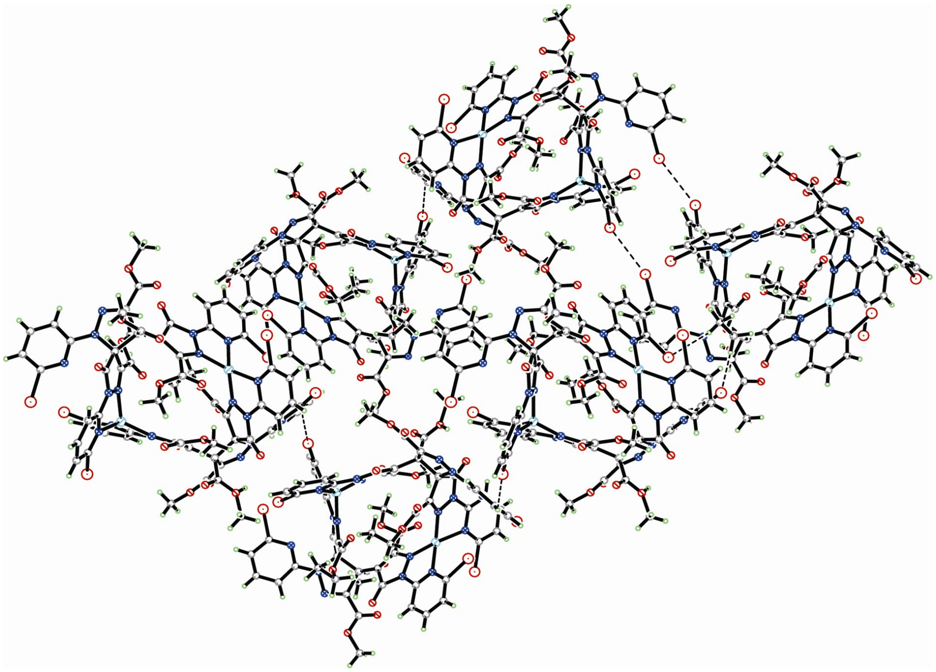

Figure S6. Packing drawing of **4**.

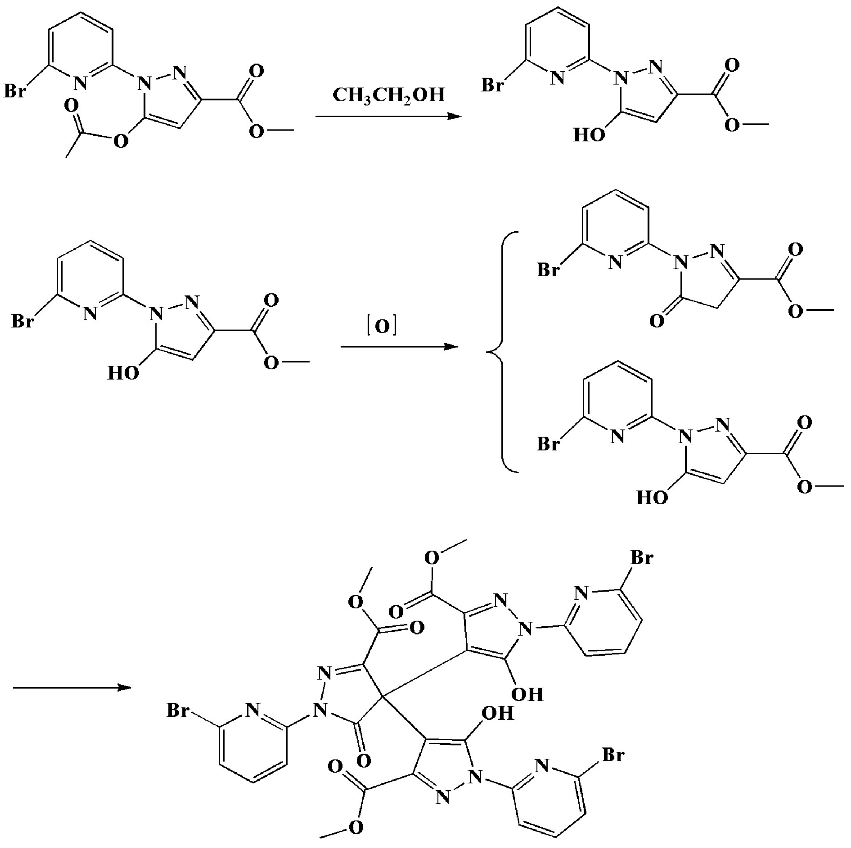

Scheme S1. Possible reaction mechanism for compound **3**.

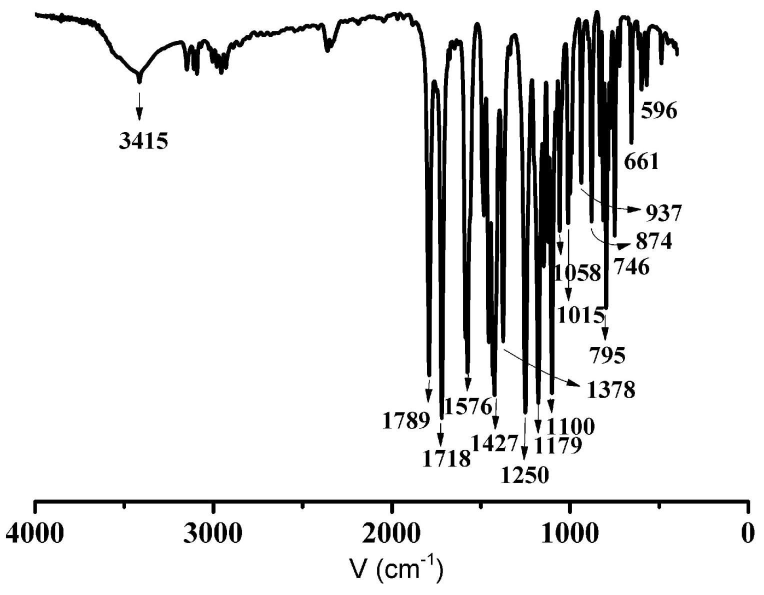

Figure S7. IR of **1**.

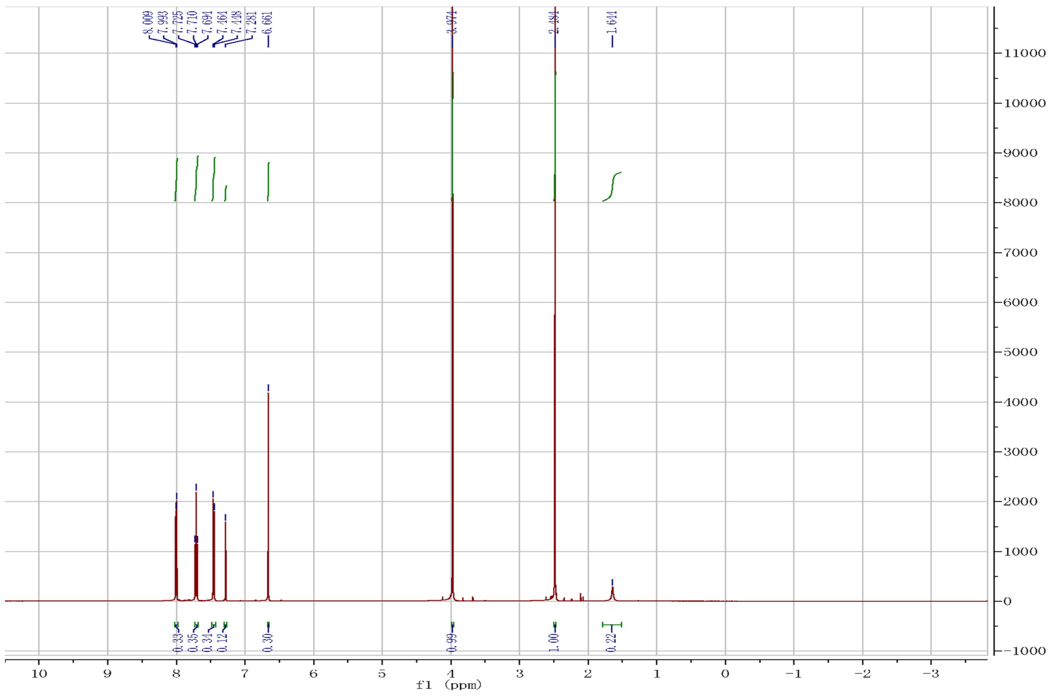

Figure S8. ^{1}H NMR of **1**.

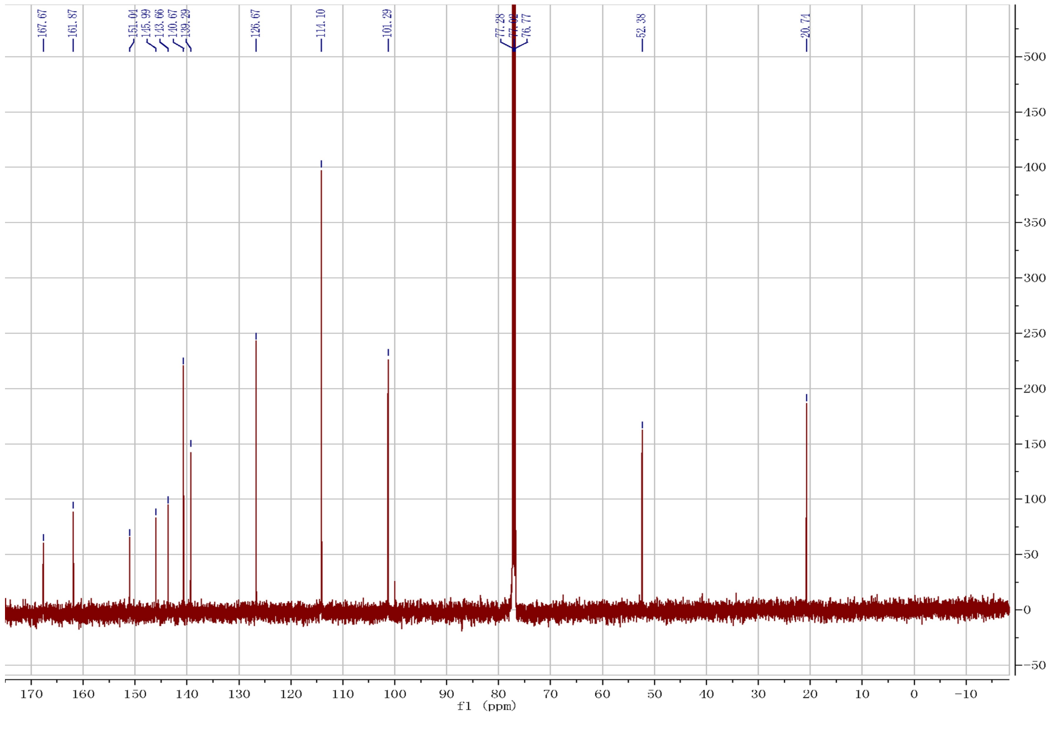

Figure S9. ^{13}C NMR of **1**.

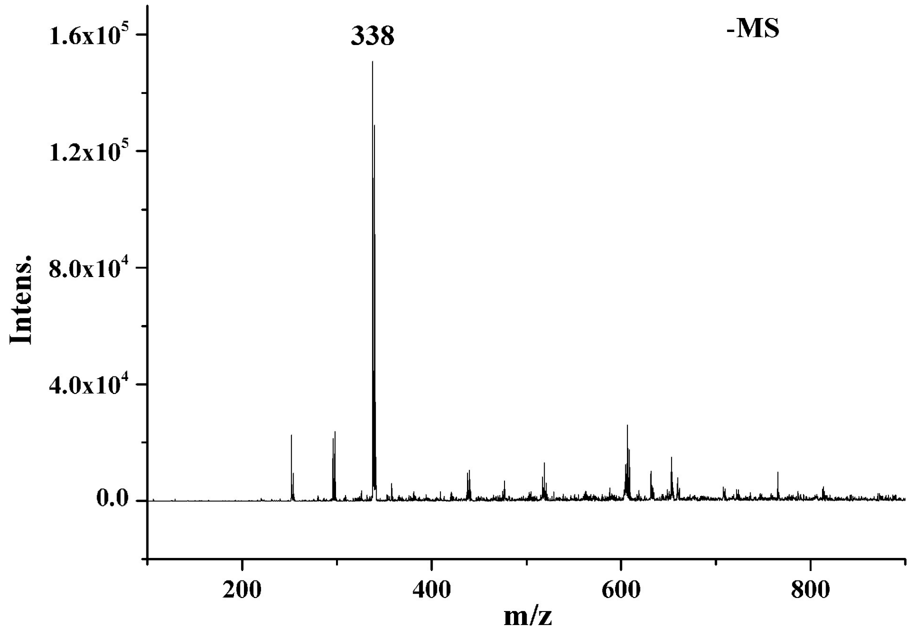

Figure S10. ESI-MS of **1**.

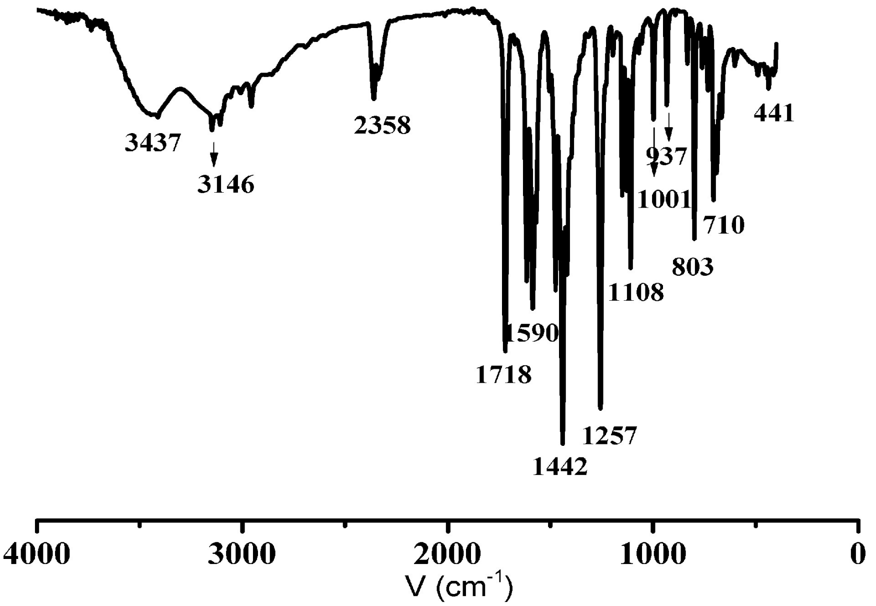

Figure S11. IR of **2**.

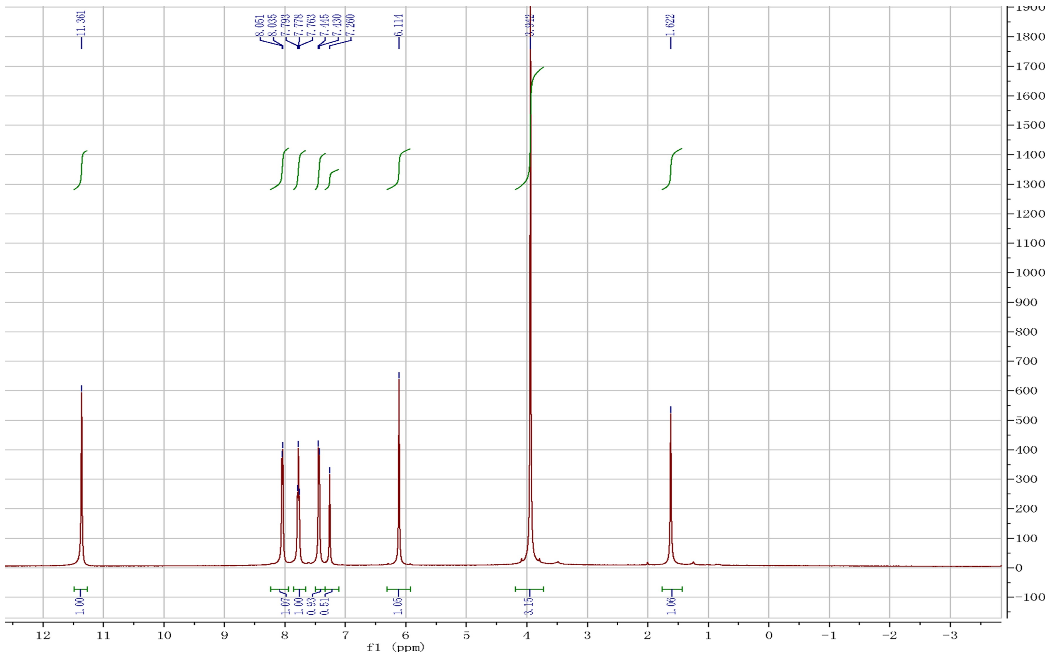

Figure S12. ^{1}H NMR of **2**.

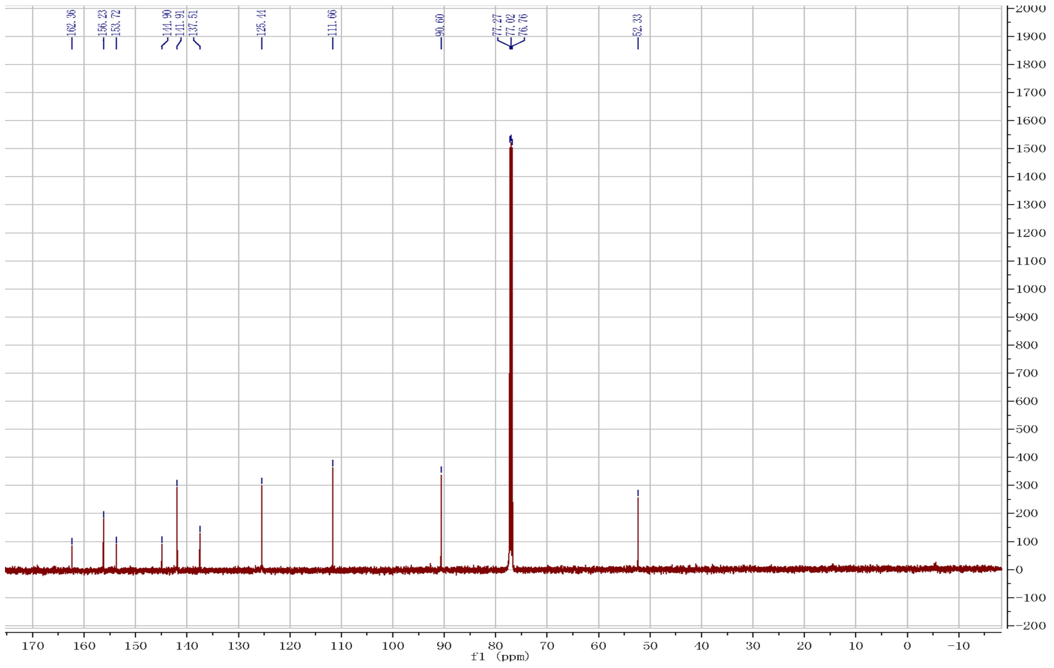

Figure S13. ^{13}C NMR of **2**.

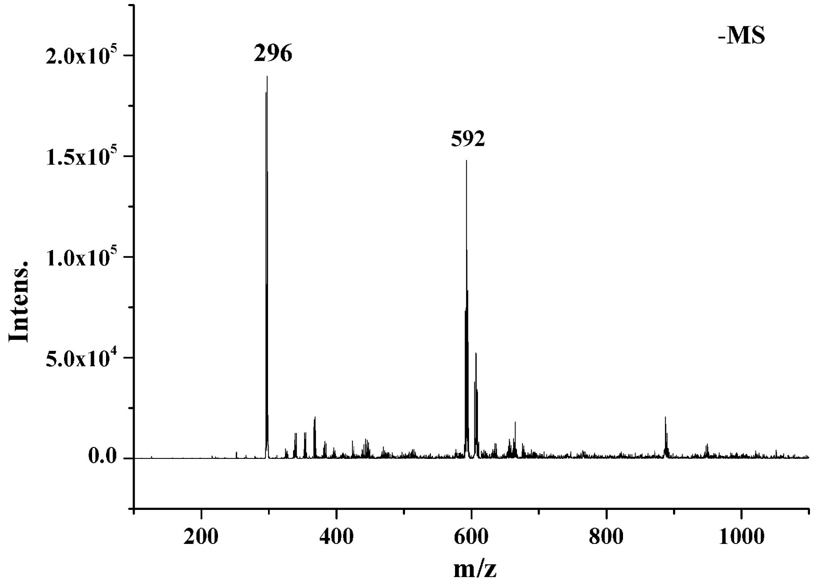

Figure S14. ESI-MS of **2**.

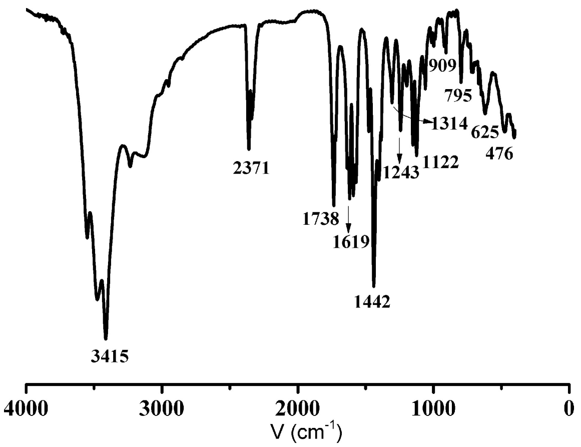

Figure S15. IR of **3**.

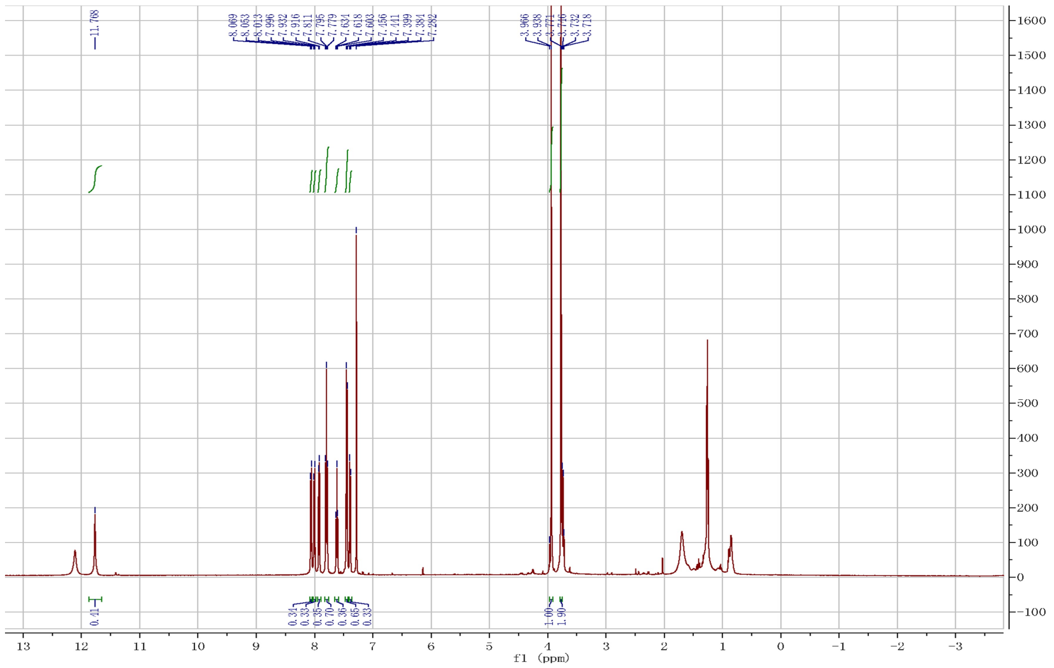

Figure S16. ^{1}H NMR of **3**.

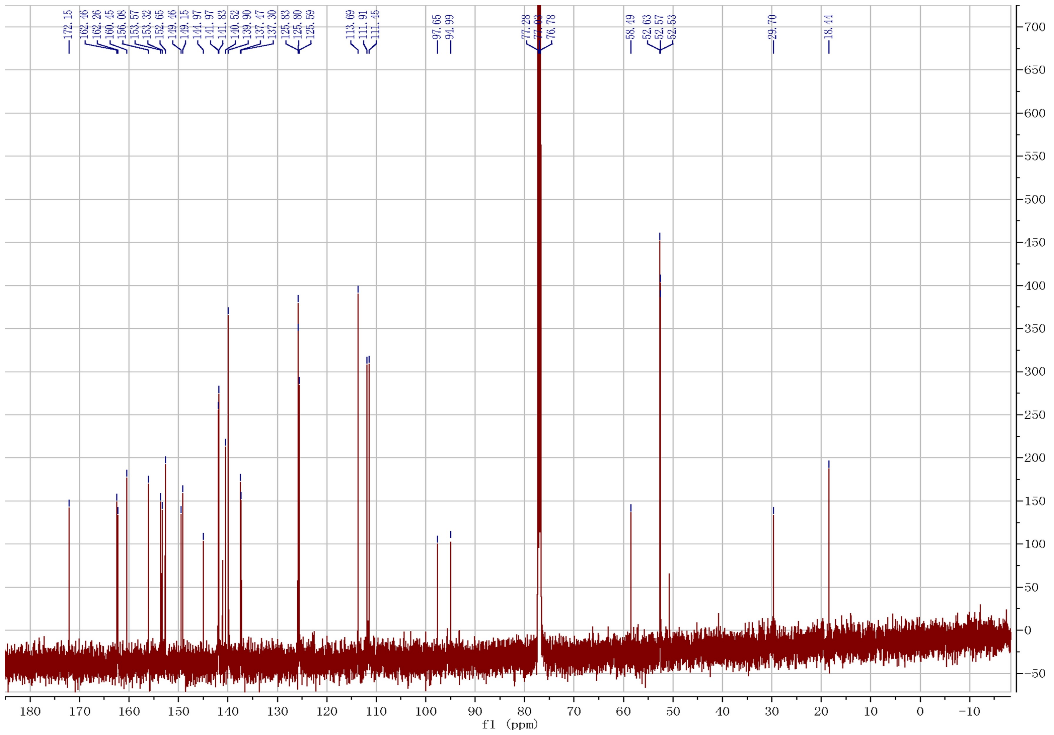

Figure S17. ^{13}C NMR of **3**.

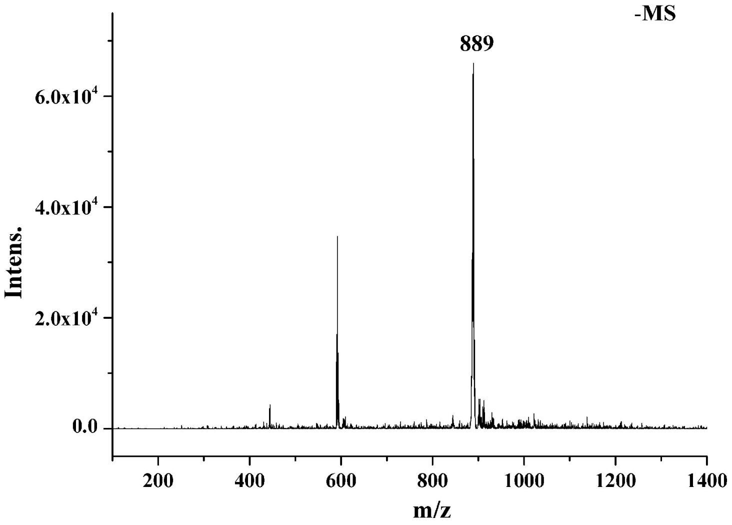

Figure S18. ESI-MS of **3**.

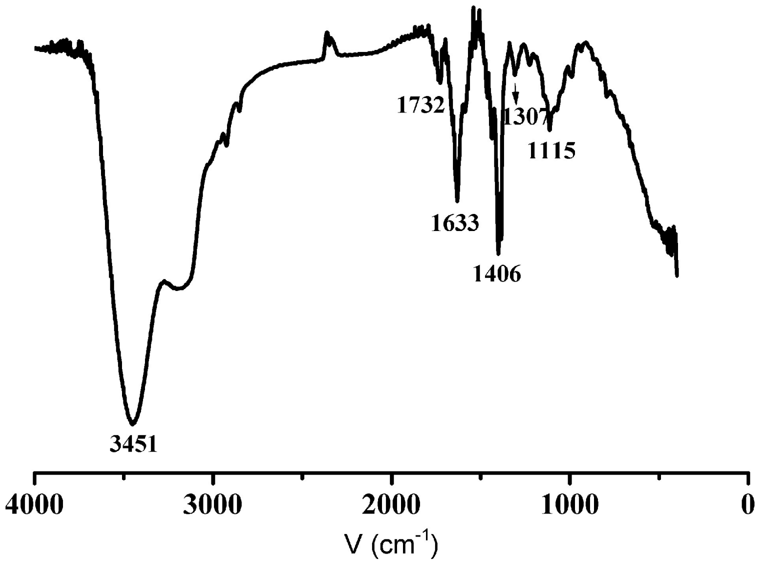

Figure S19. IR of **4**.

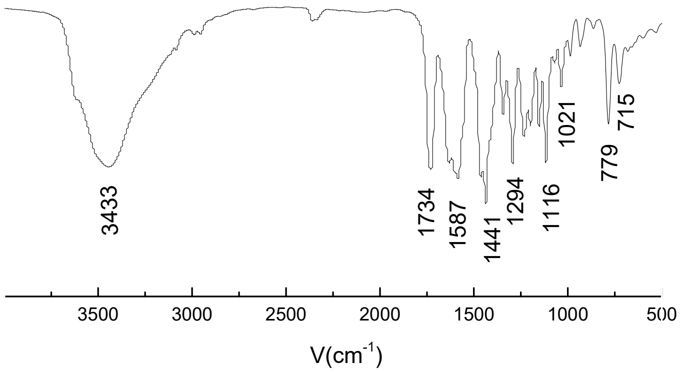

Figure S20. IR of **5**.

Table S1. Selected bond lengths (Å) and bond angles (°) for compound **1**.

O2–C9	1.393 (5)	O3–C11	1.193 (5)
O2–C6	1.359 (4)	C9–C10	1.474 (6)
O1–C9	1.187 (5)	C7–C8	1.408 (5)
O4–C11	1.323 (5)	C7–C6	1.356 (5)
O4–C12	1.446 (5)	C8–C11	1.479 (5)
N2–N3	1.358 (4)	N2–C5	1.422 (5)
N2–C6	1.371 (5)	N3–C8	1.323 (5)
O3–C11–O4	124.8 (4)	O2–C6–N2	123.2 (4)
O3–C11–C8	123.0 (4)	O2–C9–C10	109.8 (4)
C6–N2–C5	130.8 (3)	O1–C9–O2	120.9 (4)
C8–N3–N2	104.7 (3)	O1–C9–C10	129.2 (4)
N3–C8–C11	122.8 (4)	C6–C7–C8	104.3 (4)
C7–C8–C11	124.8 (4)	N3–C8–C7	112.4 (4)
O4–C11–C8	112.3 (4)	C7–C6–O2	128.8 (4)
C6–O2–C9	116.7 (3)	C7–C6–N2	107.7 (3)
C11–O4–C12	116.4 (3)	N1–C5–N2	115.8 (4)
N3–N2–C6	110.9 (3)	C4–C5–N2	120.2 (4)
N3–N2–C5	118.1 (3)		

Table S2. Selected bond lengths (Å) and bond angles (°) for compound **2**.

O3–C9	1.321 (4)	N3–N2–C6	111.3 (2)
O3–C10	1.450 (4)	C4–C5–N2	122.0 (3)
N2–C5	1.410 (4)	N1–C5–N2	114.1 (3)
N2–C6	1.373 (4)	N3–C8–C9	122.0 (3)
N2–N3	1.371 (3)	N3–C8–C7	113.1 (3)
O1–C6	1.338 (4)	C7–C8–C9	124.8 (3)
C8–C9	1.481 (4)	O1–C6–N2	123.1 (3)
C8–N3	1.319 (4)	O1–C6–C7	129.7 (3)
C8–C7	1.404 (4)	C7–C6–N2	107.3 (3)
C6–C7	1.348 (4)	O3–C9–C8	113.1 (3)
C9–O2	1.202 (4)	O2–C9–O3	125.0 (3)
C9–O3–C10	116.1 (2)	O2–C9–C8	122.0 (3)
C6–N2–C5	128.0 (3)	C8–N3–N2	103.5 (2)
N3–N2–C5	120.6 (2)	C6–C7–C8	104.7 (3)

Table S3. Selected bond lengths (Å) and bond angles (°) for compound **3**.

O5–C13	1.326 (9)	O4–C13–O5	125.4 (8)
O5–C14	1.473 (11)	O4–C13–C12	122.4 (7)
O4–C13	1.183 (9)	N4–C12–C13	120.2 (7)
O6–C15	1.304 (9)	N4–C12–C11	113.4 (7)
O9–C25	1.327 (10)	C11–C12–C13	126.5 (7)
O3–C10	1.209 (10)	C6–N3–N2	109.2 (7)
O1–C7	1.206 (11)	C20–N6–C16	115.1 (8)
N4–N5	1.376 (9)	N1–C5–N2	114.3 (7)
N4–C12	1.311 (9)	C4–C5–N2	121.0 (7)
N8–C25	1.380 (11)	C22–O8–C23	119.1 (10)
N8–N7	1.385 (11)	N3–C6–C9	113.6 (7)
N8–C26	1.425 (11)	N3–C6–C7	118.3 (8)
O2–C7	1.316 (11)	C7–C6–C9	128.1 (7)
O2–C8	1.453 (12)	C25–C24–C9	126.7 (8)
N5–C16	1.411 (10)	C25–C24–C21	106.0 (7)
N5–C15	1.365 (9)	C21–C24–C9	127.1 (7)
O7–C22	1.181 (11)	C6–C9–C24	110.1 (6)
N2–N3	1.384 (9)	C6–C9–C10	99.1 (6)
N2–C5	1.429 (10)	C11–C9–C6	116.0 (6)
N2–C10	1.380 (10)	C11–C9–C24	112.5 (6)
C13–C12	1.479 (11)	C11–C9–C10	115.0 (6)
C12–C11	1.416 (10)	C10–C9–C24	102.6 (6)
N3–C6	1.248 (10)	N6–C16–N5	114.2 (7)
O8–C22	1.328 (13)	C17–C16–N5	120.5 (8)
O8–C23	1.505 (16)	C12–C11–C9	129.8 (7)
C6–C9	1.519 (11)	C15–C11–C12	103.8 (6)
C6–C7	1.491 (12)	C15–C11–C9	126.4 (7)
C24–C9	1.549 (11)	O1–C7–O2	126.8 (9)
C24–C25	1.347 (11)	O1–C7–C6	123.0 (9)
C24–C21	1.410 (12)	O2–C7–C6	110.2 (8)
C9–C11	1.502 (10)	O9–C25–N8	121.4 (7)
C9–C10	1.541 (12)	O9–C25–C24	132.2 (8)
C11–C15	1.372 (10)	C24–C25–N8	106.4 (8)
N7–C21	1.328 (11)	C21–N7–N8	103.2 (7)
C21–C22	1.487 (12)	O6–C15–N5	123.1 (7)
C13–O5–C14	114.3 (7)	O6–C15–C11	129.6 (7)
C12–N4–N5	103.9 (6)	N5–C15–C11	107.3 (7)
C25–N8–N7	111.8 (7)	O3–C10–N2	125.1 (8)
C25–N8–C26	128.2 (8)	O3–C10–C9	129.8 (8)
N7–N8–C26	120.0 (8)	N2–C10–C9	104.6 (7)
C7–O2–C8	116.9 (8)	C24–C21–C22	128.9 (8)
N4–N5–C16	120.5 (6)	N7–C21–C24	112.5 (8)

C15–N5–N4	111.6 (6)	N7–C21–C22	118.6 (8)
C15–N5–C16	127.9 (7)	O7–C22–O8	123.9 (9)
N3–N2–C5	120.8 (6)	O7–C22–C21	125.5 (10)
C10–N2–N3	112.5 (7)	O8–C22–C21	110.6 (9)
C10–N2–C5	126.2 (7)	N9–C26–N8	113.5 (8)
O5–C13–C12	112.1 (7)	C27–C26–N8	121.2 (10)

Table S4. Selected bond lengths (Å) and bond angles (°) for compound **4**.

Cu1–N16	1.979 (8)	N16–Cu1–N3	127.7 (3)
Cu1–N18	2.078 (9)	N18–Cu1–N1	126.1 (4)
Cu1–N1	2.105 (8)	N3–Cu1–N18	122.9 (3)
Cu1–N3	2.026 (8)	N3–Cu1–N1	77.2 (3)
Cu2–N7	1.976 (9)	N7–Cu2–N12	129.7 (3)
Cu2–N12	2.004 (8)	N7–Cu2–N9	80.2 (3)
Cu2–N9	2.064 (9)	N7–Cu2–N10	124.3 (4)
Cu2–N10	2.073 (9)	N12–Cu2–N9	122.7 (3)
N16–Cu1–N18	80.1 (3)	N12–Cu2–N10	79.7 (3)
N16–Cu1–N1	130.1 (3)	N9–Cu2–N10	127.3 (3)

Table S5. Selected bond lengths (Å) and bond angles (°) for compound **5**.

Br3–C21	1.862(7)	O10–C30	1.331(7)
Br2–C11	1.859(8)	O10–C31	1.447(8)
N4–C11	1.321(8)	N4–C15	1.332(8)
O4–C16	1.187(7)	O8–C28	1.309(8)
O8–C29	1.459(8)	O1–C6	1.351(8)
N5–N6	1.367(7)	N5–C16	1.389(8)
N5–C15	1.427(7)	N9–C27	1.280(8)
N9–N8	1.372(7)	O5–C19	1.181(8)
O9–C30	1.183(8)	N6–C18	1.291(8)
C18–C19	1.489(8)	C18–C19	1.522(8)
N7–C21	1.326(9)	N7–C25	1.333(8)
O7–C28	1.211(8)	N1–C5	1.301(10)
N1–C1	1.334(9)	N3–C8	1.312(8)
N3–N2	1.354(8)	O2–C9	1.183(8)
C27–C28	1.479(8)	C27–C26	1.529(8)
N2–C6	1.366(8)	N2–C5	1.428(8)
C19–O6	1.343(8)	N8–C25	1.395(8)
N8–C17	1.478(7)	C16–C17	1.559(8)
C30–C26	1.564(8)	C15–C14	1.370(9)
C5–C4	1.401(10)	C17–C26	1.575(8)
C26–C7	1.528(8)	O6–C20	1.451(9)
O3–C9	1.329(8)	O3–C10	1.457(10)

C8–C7	1.433(8)	C8–C9	1.486(9)
C11–C12	1.365(10)	C25–C24	1.388(9)
C1–C2	1.373(11)	C1–Br1	1.830(8)
C24–C23	1.390(10)	C7–C6	1.369(8)
C22–C21	1.360(10)	C22–C23	1.376(10)
C12–C13	1.384(10)	C13–C14	1.388(9)
C3–C2	1.372(13)	C3–C4	1.376(11)
C33–N10	1.111(19)	C33–C32	1.41(2)
C30–O10–C31	115.5(5)	C11–N4–C15	115.3(6)
C28–O8–C29	115.7(6)	N6–N5–C16	114.2(4)
N6–N5–C15	118.8(4)	C16–N5–C15	126.6(5)
C27–N9–N8	108.5(5)	C18–N6–N5	108.6(4)
N6–C18–C19	121.7(5)	N6–C18–C17	113.3(5)
C19–C18–C17	124.9(5)	C21–N7–C25	116.1(5)
C5–N1–C1	116.5(6)	C8–N3–N2	104.8(5)
N9–C27–C28	122.5(5)	N9–C27–C26	115.0(5)
C28–C27–C26	122.0(5)	N3–N2–C6	111.8(5)
N3–N2–C5	120.8(5)	C6–N2–C5	127.4(6)
O5–C19–O6	126.5(6)	O5–C19–C18	122.7(6)
O6–C19–C18	110.8(5)	N9–N8–C25	120.1(5)
N9–N8–C17	112.8(4)	C25–N8–C17	122.4(5)
O4–C16–N5	127.0(5)	O4–C16–C17	128.5(5)
N5–C16–C17	104.4(4)	O9–C30–O10	126.2(5)
O9–C30–C26	124.8(5)	O10–C30–C26	108.9(5)
N4–C15–C14	124.9(5)	N4–C15–N5	114.3(5)
C14–C15–N5	120.8(5)	O7–C28–O8	126.2(6)
O7–C28–C27	120.2(5)	O8–C28–C27	113.5(5)
N1–C5–C4	125.3(6)	N1–C5–N2	115.8(6)
C4–C5–N2	118.9(6)	N8–C17–C18	112.6(5)
N8–C17–C16	113.1(4)	C18–C17–C16	99.3(4)
N8–C17–C26	101.4(4)	C18–C17–C26	112.8(4)
C16–C17–C26	118.1(5)	C7–C26–C27	113.2(5)
C7–C26–C30	110.7(5)	C27–C26–C30	106.5(4)
C7–C26–C17	113.1(4)	C27–C26–C17	99.5(4)
C30–C26–C17	113.2(4)	C19–O6–C20	114.7(6)

C9–O3–C10	114.5(6)	N3–C8–C7	112.6(5)
N3–C8–C9	119.1(5)	C7–C8–C9	128.1(6)
N4–C11–C12	126.5(7)	N4–C11–Br2	114.2(5)
C12–C11–Br2	119.2(5)	N7–C25–C24	124.3(6)
N7–C25–N8	112.4(5)	C24–C25–N8	123.3(6)
O2–C9–O3	125.2(6)	O2–C9–C8	124.5(6)
O3–C9–C8	110.3(6)	N1–C1–C2	124.2(8)
N1–C1–Br1	117.2(6)	C2–C1–Br1	118.6(6)
C25–C24–C23	116.3(6)	C6–C7–C8	103.1(5)
C6–C7–C26	127.1(5)	C8–C7–C26	129.8(5)
O1–C6–N2	121.8(5)	O1–C6–C7	130.5(5)
N2–C6–C7	107.7(5)	C21–C22–C23	116.8(6)
N7–C21–C22	125.7(6)	N7–C21–Br3	114.4(5)
C22–C21–Br3	119.9(5)	C11–C12–C13	116.1(6)
C12–C13–C14	120.2(6)	C15–C14–C13	116.9(6)
C2–C3–C4	120.4(7)	C22–C23–C24	120.7(6)
N10–C33–C32	177(2)	C3–C4–C5	115.9(7)
C3–C2–C1	117.6(7)		

Table S6. The Inhibition rates of compounds **1-5** towards different cell lines after incubation for 48 h.

complexes	BEL-7404	HepG2	NCI-H460	T-24	A549	HL-7702
**1**	38.16±1.99	47.21±0.44	33.98±1.76	37.58±1.72	31.14±1.39	26.47±0.42
**2**	20.17±1.18	43.05±0.74	38.04±0.62	42.54±0.96	22.35±1.74	24.68±0.59
**3**	24.65±0.75	41.02±1.27	42.55±2.27	31.99±0.89	33.33±2.51	22.16±1.05
**4**	39.61±1.43	53.27±0.92	33.59±1.38	43.25±1.15	40.25±0.44	35.08±1.34
**5**	27.22±0.94	19.54±1.05	25.36±0.34	35.88±1.26	29.45±0.65	26.88±0.79
**cisplatin**	55.15±1.18	60.63±0.99	50.88±3.69	47.58±2.65	60.63±0.99	73.58±2.30

Noted: The inhibition ratios for complexes **1-5** were obtained at 20 μM.

Table S7. Crystal data and structure refinements for compounds **1-5**.

Compounds	**1**	**2**	**3**	**4**	**5**
Formula	$C_{12}H_{10}N_3O_4Br$	$C_{10}H_8N_3O_3Br$	$C_{30}H_{20}N_9O_9Br_3$	$C_{63}H_{46}Cu_2N_{18}O_{21}Br_6$	$C_{33}H_{26}Br_3N_{10}O_{10}$
Form. weight	340.14	298.09	890.25	1997.72	962.34

Colour and form	block, white	block, white	block, white	block, red	block, white
T / K	293(2)	293(2)	293(2)	293(2)	293(2)
Crystal system	Triclinic	Monoclinic	Triclinic	Monoclinic	triclinic
Space group	$P\bar{1}$	$P2_1/c$	$P\bar{1}$	$P2_1/c$	$P\bar{1}$
a / Å	8.230(1)	14.198(1)	11.582(1)	14.377(1)	9.986(1)
b / Å	8.282(1)	5.131(1)	12.322(1)	22.407(1)	11.569(1)
c / Å	10.214(1)	15.229(1)	15.877(1)	23.662(2)	17.669(1)
α / °	75.47(1)	90.00	83.66(1)	90.00	84.55(1)
β / °	85.27(1)	99.74(1)	71.06(1)	96.98(1)	88.84(1)
γ / °	85.32(1)	90.00	65.39(1)	90.00	64.57(1)
V / Å^3	670.3(1)	1093.4(2)	1947.7(3)	7565.8(9)	1834.5(1)
Z	2	4	2	4	2
D_{calcd}/gcm^{-3}	1.685	1.805	1.518	1.754	1.742
μ / mm^{-1}	3.083	3.755	3.165	3.814	3.371
R_{int}	0.0256	0.0371	0.0400	0.0819	0.0210
Goof	0.997	1.008	1.008	1.004	1.004
Completeness	99.7%	99.7%	99.5%	99.8%	98.3%
F(000)	340	592	880	3880	956
θ range / °	3.13 to 26.37	2.91 to 26.37	2.95 to 25.01	2.85 to 25.01	3.27 to 25.00
Ref.coll. / unique	4558 / 2733	5313 / 2228	13596 / 6833	30397 / 13245	10865 / 6344
Parameters	183	158	469	998	506
Final R_1 [$I > 2\sigma$][a]	0.0485	0.0405	0.0723	0.0852	0.0908
wR_2[b]	0.1217	0.0898	0.2339	0.2436	0.2780
Residues / eÅ^{-3}	0.375, −0.541	0.306, −0.456	0.980, −0.577	1.388, −0.912	0.944, -0.929

[a] $R_1 = \Sigma||F_o| - |F_c||/\Sigma|F_o|$. [b] $wR_2 = [\Sigma w(|F_o^2| - |F_c^2|)^2/\Sigma w(|F_o^2|)^2]^{1/2}$.
